# Loss of MNX1 Sensitizes Tumors to Cytotoxic T Cells by Degradation of PD‐L1 mRNA

**DOI:** 10.1002/advs.202403077

**Published:** 2025-02-06

**Authors:** Zhengzheng Li, Lei Chen, Ge Zhang, Shuang Wang, Enhang Xu, Jinglei Teng, Jiancheng Xu, Fang Peng, Qingjie Min, Zhuoya Wang, Shujuan Shao, Lianmei Zhao, Baoen Shan, Yang Wang, Qimin Zhan, Xuefeng Liu

**Affiliations:** ^1^ Institute of Cancer Stem Cell Dalian Medical University Dalian 116044 China; ^2^ Soochow University Cancer Institute Suzhou 215000 China; ^3^ Department of Pulmonary Oncology Affiliated Hospital of Guangdong Medical University Zhanjiang 524001 China; ^4^ Department of Immunology College of Basic Medical Sciences Dalian Medical University Dalian 116044 China; ^5^ Department of Pathology the Second Affiliated Hospital of Dalian Medical University Dalian 116023 China; ^6^ University Key Laboratory of Proteomics in Liaoning Province Dalian Medical University Dalian 116044 China; ^7^ Research Center the Fourth Hospital of Hebei Medical University Shijiazhuang 050011 China; ^8^ Key Laboratory of Carcinogenesis and Translational Research (Ministry of Education/Beijing) Laboratory of Molecular Oncology Peking University Cancer Hospital & Institute Beijing 100142 China

**Keywords:** immunotherapy, MNX1, MNX1‐AS1, PD‐L1, tumor immune escape

## Abstract

Immune checkpoint blockade (ICB) therapy, targeting programmed cell death ligand‐1 (PD‐L1)/programmed cell death protein 1 (PD‐1) axis and cytotoxic T‐lymphocyte‐associated protein 4 (CTLA‐4), has exhibited amazing clinical outcomes in various types of cancers. However, only a small portion of patients benefit from ICB therapy, indicating that the mechanism underlying immune checkpoint is still unclear. Here, it is reported that motor neuron and pancreas homeobox 1 (MNX1), a homeobox domain‐containing transcription factor, contributes to the tumor immune escape. MNX1 increases PD‐L1 expression in cancer cells by stabilizing PD‐L1 mRNA rather than activating transcription. Mechanistically, MNX1 exists in the cytoplasm of cancer cells and interacts with Y‐box binding protein 1 (YBX1), a multifunctional DNA/RNA‐binding protein, to enhance the binding of YBX1 to PD‐L1 mRNA. MNX1 ablation activates cytotoxic T cell‐mediated anti‐tumor immunity and sensitizes CTLA‐4 blockade therapy. Moreover, MNX1 also facilitates tumor progression in an immune‐independent manner in cancer cells. In addition, MNX1 is upregulated by its adjacent long non‐coding RNA MNX1‐AS1 via HECT and RLD domain containing E3 ubiquitin protein ligase 2 (HERC2). Together, these results reveal MNX1 as a novel immune checkpoint regulator with promising therapeutic potential.

## Graphical abstract

This study presents motor neuron and pancreas homeobox 1 (MNX1)‐mediated programmed cell death ligand‐1 (PD‐L1) mRNA stability in cancer cells as a novel immunosuppressive mechanism. MNX1, stabilized by MNX1‐AS1, binds to YBX1 to enhance PD‐L1 mRNA stability. Loss of MNX1 enhances T cell anti‐tumor immunity and cytotoxic T‐lymphocyte‐associated protein 4 (CTLA‐4) blockade therapy, providing a potential strategy for cancer immunotherapy.

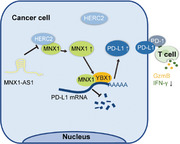

## 1 Introduction

Cancers gradually acquire immunosuppressive mechanisms during disease progression to evade the immune surveillance.^[^ ^1^ ^]^ The activation of immune checkpoint pathways is a critical mechanism of tumor immune escape. Binding of programmed cell death ligand‐1 (PD‐L1) expressed on tumor cells to programmed cell death protein 1 (PD‐1) on activated T cells creates a major inhibitory immune checkpoint to suppress the T cell anti‐tumor function.^[^ ^2^, ^3^ ^]^ Therefore, blocking immune checkpoints, especially PD‐L1/PD‐1 axis, has shown encouraging clinical outcomes across numerous cancer types, such as melanoma, non‐small cell lung cancer, breast cancer, as well as esophageal cancer (ESCA).^[^ ^4^, ^5^, ^6^, ^7^, ^8^ ^]^ However, in many scenarios, only a subset of patients benefits from immune checkpoint blockade (ICB) therapy and some patients can even develop acquired resistance,^[^ ^9^, ^10^ ^]^ suggesting that the mechanisms of immune checkpoint pathways are not fully understood.

PD‐L1 expression is a widely used biomarker to predict the response to PD‐1 or PD‐L1 inhibitors.^[^ ^11^, ^12^ ^]^ PD‐L1 can be induced by inflammatory cytokines produced in the tumor microenvironment, such as interferon‐gamma (IFN‐γ) and tumor necrosis factor‐alpha (TNF‐α).^[^ ^2^, ^13^, ^14^ ^]^ Oncogenic pathways,^[^ ^15^, ^16^ ^]^ genetic alteration,^[^ ^17^, ^18^, ^19^ ^]^ epigenetic modification,^[^ ^20^, ^21^ ^]^ as well as post‐translation modification^[^ ^22^, ^23^ ^]^ are also regulatory mechanisms of PD‐L1 expression in cancer. Additionally, the stability of PD‐L1 mRNA is involved in regulation of PD‐L1 expression as well. Besides microRNA‐mediated degradation of PD‐L1 mRNA, RNA‐binding proteins human antigen R (HuR) and GTPase‐activating protein (SH3 domain)‐binding protein 2 (G3BP2) have been reported to bind to and stabilize PD‐L1 mRNA, thus increasing PD‐L1 expression.^[^ ^24^, ^25^ ^]^ However, the mechanisms that regulate PD‐L1 expression remain unclear, and further insight into the regulatory mechanisms of PD‐L1 expression will benefit the discovery of new therapeutic targets and enhance the efficacy of PD‐L1/PD‐1 blockade or other ICBs.

Motor neuron and pancreas homeobox 1 (MNX1), also named as HB9 or HLXB9, encodes a homeobox domain‐containing transcription factor. MNX1 was originally found to be expressed in lymphoid and pancreatic tissues.^[^ ^26^ ^]^ It was later identified as an important developmental gene involved in pancreatic and motor neuronal differentiation.^[^ ^27^, ^28^, ^29^ ^]^ Increasing evidence indicates that MNX1 is overexpressed in multiple types of human cancers and contributes to cancer progression.^[^ ^30^, ^31^, ^32^, ^33^, ^34^, ^35^, ^36^, ^37^ ^]^ Here, we demonstrated that MNX1, in addition to being an oncogene, also plays a crucial role in tumor immune escape, promoting tumor progression in both immune‐dependent and non‐immune‐dependent ways. Interestingly, transcription factor MNX1 exhibits cytoplasmic localization characteristic in cancer cells and stabilizes PD‐L1 mRNA by interacting with Y‐box binding protein 1 (YBX1) and increasing YBX1 binding to PD‐L1 mRNA. Depletion of MNX1 stimulates the cytotoxic T cell‐mediated anti‐tumor immunity and enhances the therapy effect of cytotoxic T‐lymphocyte‐associated protein 4 (CTLA‐4) blockade. Thus, our results reveal a new function of MNX1 beyond the transcriptional regulation and identified MNX1 as a potential target for cancer immunotherapy.

## 2 Results

### 2.1 MNX1 Correlates with Cancer Immune Response

**Figure 1:**
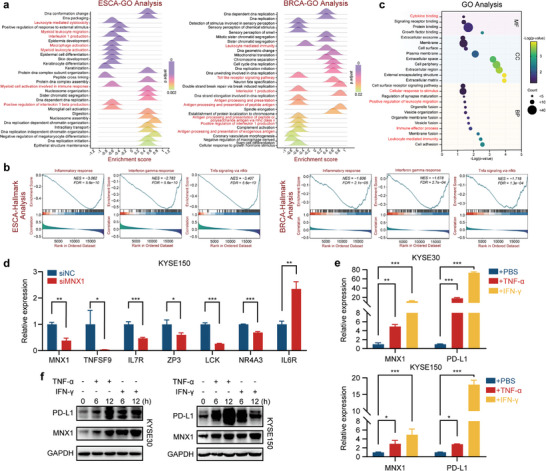
MNX1 correlates with cancer immune response. a,b) Gene set enrichment analysis (GSEA) showing MNX1‐associated functions and pathways in ESCA and BRCA, based on BEST database. For (a), data were obtained from BEST database and images were drawn through ChiPlot online website (https://www.chiplot.online/). c) GO analysis of DEGs between KYSE150 cells transfected with MNX1 siRNA or negative control siRNA, based on RNA‐seq data. d) The expression of representative immune‐related genes in KYSE150 cells transfected with siRNAs as shown in (c), validated by RT‐qPCR. e,f) The expression of MNX1 and PD‐L1 in ESCC cells stimulated with TNF‐α (20 ng mL^−1^) or IFN‐γ (20 ng mL^−1^), as determined by RT‐qPCR (e, treated for 12 h) and western blotting (f, treated for 6 and 12 h). The data are presented as mean ± SD (*n* = 3). The *p* value was calculated by two‐tailed unpaired Student's *t*‐test in (d) and by two‐way ANOVA in (e). *p < 0.05; **p < 0.01; ***p < 0.001.

To comprehensively understand the role of MNX1 in cancer, we visited the Biomarker Exploration for Solid Tumors (BEST, https://rookieutopia.com/) website^[^ ^38^ ^]^ and conducted functional and pathway enrichment analyses. Interestingly, the analyses showed that MNX1 was closely associated with immune‐related functions and pathways in multiple types of human cancers, including ESCA, breast invasive carcinoma (BRCA), colorectal cancer (CRC), and lung adenocarcinoma (LUAD) (**Figure** **1**a,b and Figure S1a,b in Supporting Information). Esophageal squamous cell carcinoma (ESCC) is the most prevalent subtype of ESCA and carries a poor prognosis, with China representing approximately half of the newly diagnosed cases.^[^ ^39^ ^]^ In recent years, ICB‐based immunotherapy has shown a favorable effect on patients with ESCA.^[^ ^40^ ^]^ Therefore, to confirm the association of MNX1 with cancer immunity, we silenced MNX1 expression in ESCC cell line KYSE150, which had relative high expression of MNX1 (Figure 6c,d), by transfection of MNX1‐specific small interfering RNAs (siRNAs) (Figure S1c,d in Supporting Information). RNA sequencing (RNA‐seq) and Gene Ontology (GO) analysis of MNX1‐depleted KYSE150 cells and control cells revealed that depletion of MNX1 also led to an enrichment in immune‐related processes (Figure 1c). We then validated the expression of some immune‐related genes using reverse transcription‐quantitative PCR (RT‐qPCR) and observed results consistent with RNA‐seq (Figure 1d). To examine whether MNX1 could respond to inflammatory cytokines, we treated KYSE150 and KYSE30 cells with IFN‐γ and TNF‐α, respectively. As expected, MNX1 was upregulated at both mRNA and protein levels in response to IFN‐γ and TNF‐α stimulation, which was consistent with the well‐known response gene PD‐L1 (Figure 1e,f). Altogether, these results suggest that MNX1 may be involved in the regulation of tumor immune response.

### 2.2 MNX1 Regulates PD‐L1 Expression in Cancer Cells

**Figure 2:**
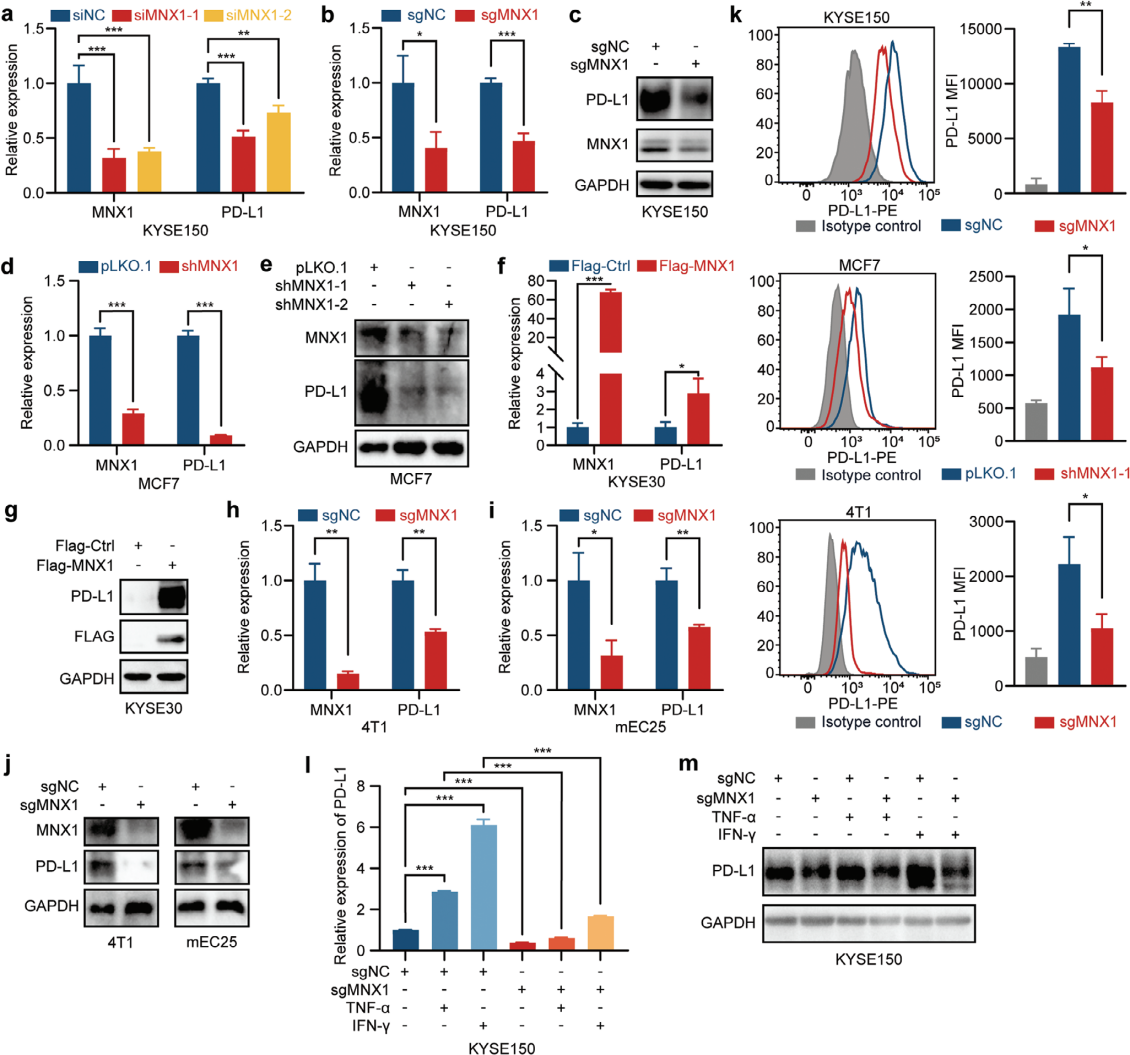
MNX1 regulates PD‐L1 expression in cancer cells. a) The mRNA levels of MNX1 and PD‐L1 in KYSE150 cells upon siRNA‐mediated MNX1 knockdown, as determined by RT‐qPCR. b) The mRNA and c) protein levels of MNX1 and PD‐L1 in KYSE150 cells upon CRISPR/Cas9‐mediated MNX1 knockout, as determined by RT‐qPCR and western blotting. d) The mRNA and e) protein levels of MNX1 and PD‐L1 in MCF7 cells upon shRNA‐mediated MNX1 knockdown, as determined by RT‐qPCR and western blotting. f) The mRNA and g) protein levels of MNX1 and PD‐L1 in KYSE30 cells upon MNX1 overexpression, as determined by RT‐qPCR and western blotting. h‐j) The mRNA and protein levels of MNX1 and PD‐L1 in 4T1 and mEC25 mouse cancer cells upon CRISPR/Cas9‐mediated MNX1 knockout, as determined by RT‐qPCR and western blotting. k) The expression of PD‐L1 on plasma membrane of KYSE150, MCF7, and 4T1 cancer cells upon MNX1 knockdown or knockout, as analyzed by flow cytometry. MFI, mean ﬂuorescence intensity. l) The mRNA and m) protein levels of PD‐L1 in MNX1‐KO KYSE150 cells and control cells stimulated with or without IFN‐γ or TNF‐α for 12 h, as determined by RT‐qPCR and western blotting. The data are presented as mean ± SD (*n* = 3). The *p* value was calculated by two‐tailed unpaired Student's *t*‐test in (b,d,f,h,i,k) and by one‐way ANOVA in (a,l). *p < 0.05; **p < 0.01; ***p < 0.001.

Based on the similar change between MNX1 and PD‐L1 in response to inflammatory cytokines, we speculated that MNX1 may regulate PD‐L1 expression in cancer cells. As expected, the depletion of MNX1, via siRNAs or clustered regularly interspaced short palindromic repeats/CRISPR‐associated protein 9 (CRISPR/Cas9) system, decreased PD‐L1 expression at both mRNA and protein levels in KYSE150 ESCC cells (**Figure** **2**a–c and Figure S1d in Supporting Information). Likewise, the depletion of MNX1 mediated by short hairpin RNA (shRNA) also led to a decrease in PD‐L1 mRNA and protein levels in MCF7 breast cancer cells (Figure 2d,e). In contrast, ectopic expression of MNX1 increased PD‐L1 expression in KYSE30 ESCC cells (Figure 2f,g). To verify this phenomenon, we subsequently examined PD‐L1 expression in MNX1‐knockout (KO) mouse tumor cells. In agreement with results from human cancer cells, the depletion of MNX1 via CRISPR/Cas9 also reduced PD‐L1 expression in 4T1 mouse breast cancer cells and mEC25 mouse ESCC cells (Figure 2h–j). Given that plasma membrane PD‐L1 on cancer cells interacts with PD‐1 on T cells to evade anti‐tumor immunity, we then employed flow cytometry to analyze the levels of PD‐L1 on plasma membrane, and observed that the membrane PD‐L1 was decreased upon MNX1 depletion in KYSE150, MCF7, and 4T1 cells (Figure 2k). Furthermore, we found that depletion of MNX1 inhibited the response of PD‐L1 to inflammatory cytokines IFN‐γ and TNF‐α (Figure 2l,m and Figure S2 in Supporting Information), indicating that MNX1 is involved in the induction of PD‐L1 by inflammatory cytokines. In summary, MNX1 can regulate the expression of PD‐L1 in tumor cells and affect the response of PD‐L1 to inflammatory cytokines.

### 2.3 MNX1 Regulates PD‐L1 mRNA Stability by Interacting with YBX1

**Figure 3:**
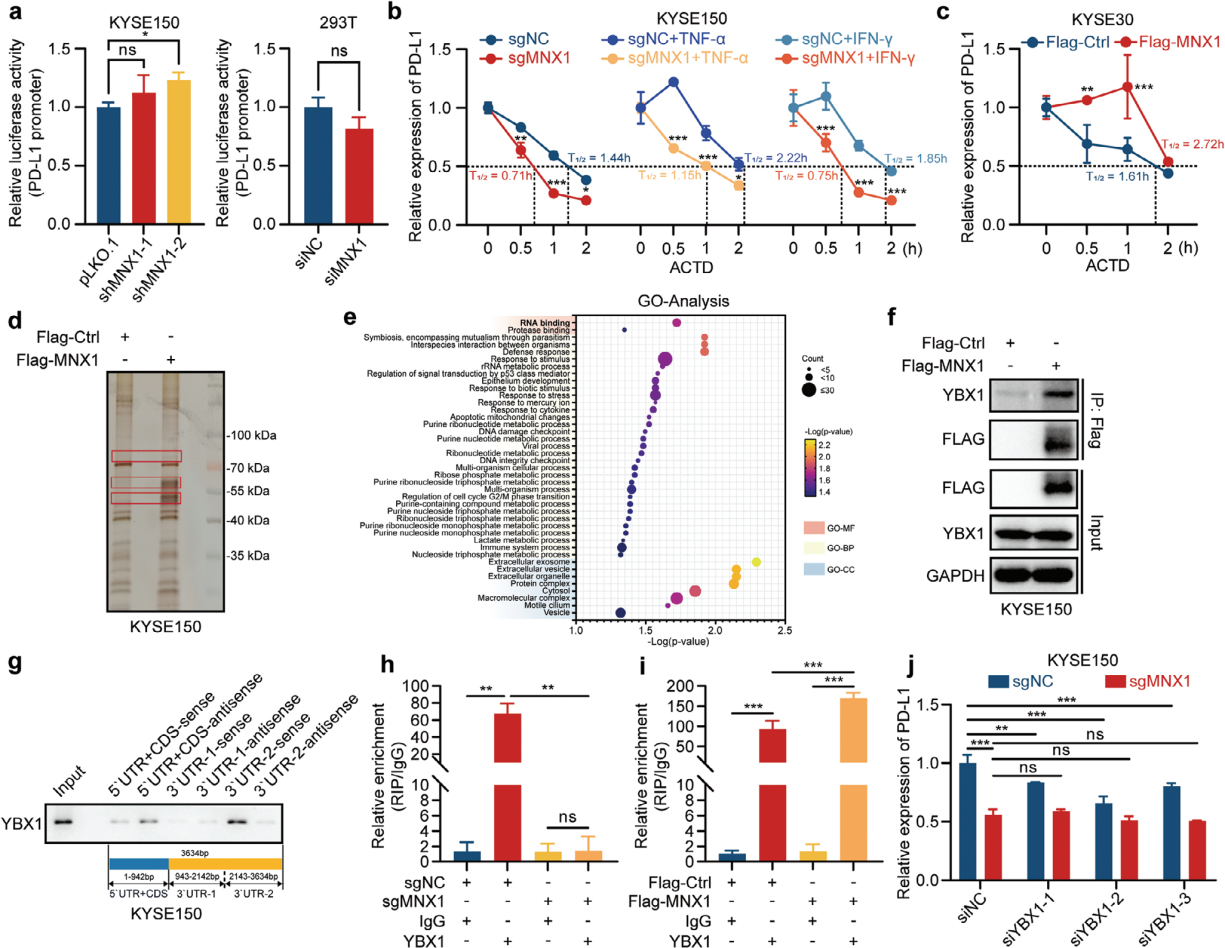
MNX1 regulates PD‐L1 mRNA stability by interacting with YBX1. a) Luciferase reporter assay showing the transcriptional activity of PD‐L1 promoter in KYSE150 (left) or HEK293T (right) cells upon MNX1 knockdown. b,c) The mRNA stability of PD‐L1 in MNX1‐KO KYSE150 cells or MNX1‐overexpressing KYSE30 cells with or without TNF‐α or IFN‐γ treatment for 12 h, detected by RT‐qPCR. Cells were treated with actinomycin D (ACTD; 5 µg mL^−1^) for the indicated time points before harvesting. d,e) IP combined with mass spectrometry analysis of the candidate MNX1‐interacting proteins. KYSE150 cells transfected with Flag‐tagged MNX1 vector or Flag empty vector were lysed and incubated with anti‐Flag M2 Affinity Gel. The retrieved proteins were subjected to SDS‐PAGE and silver staining. The bands specific to Flag‐tagged MNX1 relative to Flag control, indicated in red box (d), were analyzed by mass spectrometry. The candidate MNX1‐interacting proteins were enriched by GO analysis (e). f) Western blotting following IP validated the interaction between Flag‐tagged MNX1 and YBX1 in KYSE150 cells as shown in (d). g) RNA pull‐down assay showing the interaction between fragmented PD‐L1 mRNA and YBX1. The fragmented, biotin‐labelled PD‐L1 mRNAs or their antisenses were incubated with KYSE150 cell lysates. Western blotting was then conducted with anti‐YBX1 antibody. h,i) RIP combined with RT‐qPCR showing the interaction between YBX1 and PD‐L1 mRNA in MNX1‐KO KYSE150 cells (h) or KYSE150 cells transfected with Flag‐tagged MNX1 vector for 24 h (i). The primer used to amplify PD‐L1 mRNA was located on its 3′ UTR. The enrichment of PD‐L1 mRNA to anti‐YBX1 antibody was normalized by IgG control (*n* = 2–3). j) The mRNA levels of PD‐L1 in MNX1‐KO KYSE150 cells and control cells transfected with YBX1 siRNAs, as detected by RT‐qPCR. The data are presented as mean ± SD (*n* = 3). The *p* value was calculated by two‐tailed unpaired Student's *t*‐test in (a, right), by one‐way ANOVA in (a, left; h,i), and by two‐way ANOVA in (b,c,j). *p < 0.05; **p < 0.01; ***p < 0.001; ns, no significant.

We then set out to explore the molecular mechanisms of how MNX1 regulates PD‐L1 expression. MNX1 has been reported to be a transcription factor, so we used a pGL3 luciferase reporter vector containing the PD‐L1 promoter to examine the role of MNX1 in PD‐L1 transcriptional regulation. However, we found that depletion of MNX1 did not reduce the luciferase activity driven by PD‐L1 promoter (**Figure** **3**a and Figure S3a in Supporting Information). In addition to transcriptional regulation, mRNA stabilization is another important regulatory factor for mRNA abundance. To investigate whether MNX1 can regulate PD‐L1 mRNA stability, we treated MNX1‐KO or overexpressing ESCC cells with actinomycin D, an RNA synthesis inhibitor. As shown in Figure 3b,c, upon actinomycin D treatment, depletion of MNX1 accelerated, whereas ectopic expression of MNX1 inhibited the degradation of PD‐L1 mRNA, indicating that MNX1 can enhance the stability of PD‐L1 mRNA. We then evaluated the effect of MNX1 on PD‐L1 mRNA stability upon treatment with TNF‐α or IFN‐γ. Consistently, the depletion of MNX1 also destabilized PD‐L1 mRNA under TNF‐α or IFN‐γ treatment (Figure 3b).

Given that MNX1 does not have a classical RNA‐binding domain, we guessed that RNA‐binding proteins may be involved in the MNX1‐mediated regulation of PD‐L1 mRNA stability. To identify the MNX1‐interacting proteins, we performed immunoprecipitation (IP) assay with anti‐Flag M2 Affinity Gel in KYSE150 cells transfected with Flag‐tagged MNX1 vector or Flag empty vector, and observed several bands specific to Flag‐tagged MNX1 when compared with Flag control (Figure 3d). Mass spectrometry and GO analysis of differential bands revealed that the candidate MNX1‐interacting proteins could been significantly enriched in RNA binding (Figure 3e), of which YBX1 attracted our attention. YBX1 is a multifunctional DNA/RNA‐binding protein, which has been reported to participate in the regulation of mRNA stability.^[^ ^41^, ^42^ ^]^ Expectedly, western blotting following IP assay verified that MNX1 was able to bind to YBX1 (Figure 3f).

To determine whether YBX1 can bind to PD‐L1 mRNA, we performed RNA pull‐down assay on fragmented PD‐L1 mRNA in KYSE150 cell lysates and observed the interaction of YBX1 with the latter half of the 3′ untranslated region (UTR) of PD‐L1 mRNA (Figure 3g). RNA immunoprecipitation (RIP) assay in KYSE150 cells also showed an obvious enrichment of PD‐L1 mRNA in YBX1‐associated complex compared with IgG control; however, the depletion of MNX1 inhibited, while the ectopic expression of MNX1 promoted this enrichment (Figure 3h,i), indicating that MNX1 can enhance the interaction between YBX1 and PD‐L1 mRNA. Subsequently, we examined the impact of MNX1 on YBX1 expression, and observed no significant changes in YBX1 mRNA and protein levels following MNX1 knockout in KYSE150 cells (Figure S3b,c in Supporting Information).

To determine whether YBX1 is required for MNX1‐mediated PD‐L1 mRNA stability, we employed siRNAs to knock down YBX1 in KYSE150 cells (Figure S3d in Supporting Information). Knockdown of YBX1 also resulted in a decrease in PD‐L1 mRNA, and the inhibitory effect of MNX1 depletion on PD‐L1 expression was attenuated upon YBX1 knockdown (Figure 3j). We then overexpressed MNX1 in KYSE150 cells transfected with YBX1 siRNAs and assessed the stability of PD‐L1 mRNA. Expectedly, the knockdown of YBX1 diminished PD‐L1 mRNA stability induced by MNX1 overexpression (Figure S3e in Supporting Information), indicating that YBX1 is necessary for MNX1‐mediated PD‐L1 mRNA stability. Taken together, these results suggest that MNX1 enhances the stability of PD‐L1 mRNA by promoting the binding of YBX1 to PD‐L1 mRNA, which goes beyond its transcriptional function.

### 2.4 Depletion of MNX1 Enhances T Cell Anti‐Tumor Immunity

**Figure 4:**
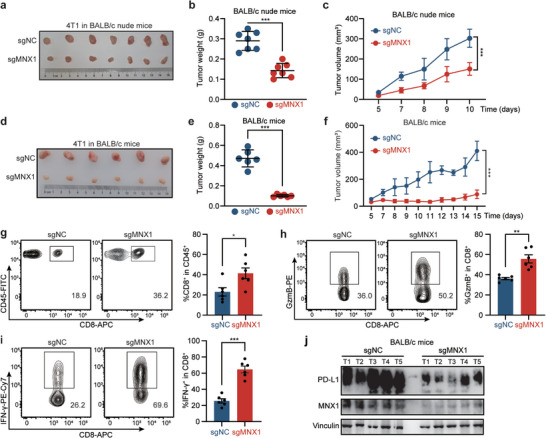
Depletion of MNX1 enhances T cell anti‐tumor immunity. a–c) MNX1‐KO 4T1 cells and control cells were injected subcutaneously into BALB/c nude mice, respectively (*n* = 7 per group). The images of established tumors (a) as well as the quantification of tumor weight (b) and volume (c) are shown. d–f) MNX1‐KO 4T1 cells and control cells were injected subcutaneously into BALB/c mice, respectively (*n* = 6 per group). The images of established tumors (d) as well as the quantification of tumor weight (e) and volume (f) are shown. g) CD8^+^ T cell populations infiltrated in tumors of BALB/c mice as well as h) GzmB‐expressing and i) IFN‐γ‐expressing CD8^+^ T cells were analyzed by flow cytometry. Representative images (left) and quantitative analyses (right) are shown (*n* = 6). j) The expression of MNX1 and PD‐L1 in tumors of BALB/c mice, as determined by western blotting. The data are presented as mean ± SD, except for (g–i) expressed as mean ± SEM. The *p* value was calculated by two‐tailed unpaired Student's *t*‐test in (b,e,g–i) and by two‐way ANOVA in (c,f). *p < 0.05; **p < 0.01; ***p < 0.001.

Based on the regulatory role of MNX1 in PD‐L1, we sought to examine whether depletion of MNX1 would affect the anti‐tumor immune response. Mouse 4T1 tumor model has been widely used in the study of anti‐tumor immune response and immunotherapy. Thus, we inoculated MNX1‐KO 4T1 cells and control cells into BALB/c nude mice (immune deficient) or BALB/c mice (immune competent), respectively. Intriguingly, the depletion of MNX1 suppressed the growth of 4T1 cells in both immune deficient and immune competent mouse models (**Figure** **4**a–f). However, the inhibitory effect is much more pronounced in immune competent model than that in immune deficient model (Figure 4a–f), indicating that the difference in tumorigenicity may be due to the immune surveillance. We then isolated lymphocytes from 4T1 tumors in immune competent mice and analyzed them by flow cytometry. As shown in Figure 4g, depletion of MNX1 increased the CD8^+^ T cell infiltration in tumors. Moreover, we also observed that MNX1 depletion led to an increase in granzyme B (GzmB)‐producing and IFN‐γ‐producing CD8^+^ T cells (Figure 4h,i). Consistently, the depletion of MNX1 in KYSE150 cells also increased the production of GzmB and IFN‐γ in co‐cultured Jurkat cells (Figure S4 in Supporting Information). Additionally, PD‐L1 expression in mouse 4T1 tumors was downregulated following the knockout of MNX1 (Figure 4j). Collectively, all of above findings suggest that MNX1 ablation activates T cell‐mediated anti‐tumor immunity via destabilizing PD‐L1 mRNA, thus inhibiting the tumor growth.

### 2.5 MNX1 Promotes Cancer Cell Malignant Characters Independently of Immunity

**Figure 5:**
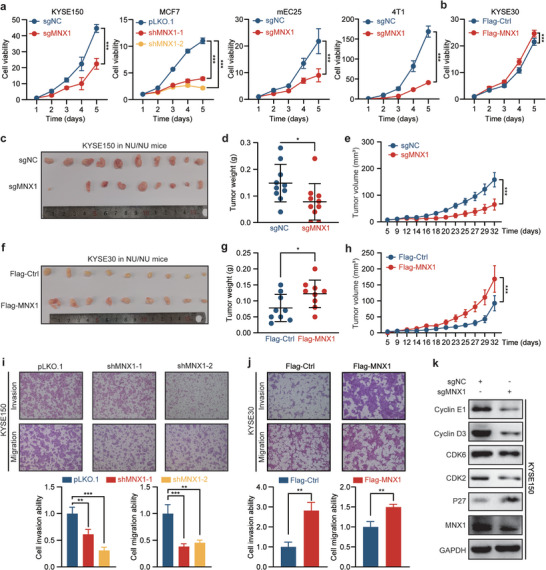
MNX1 promotes cancer cell malignant characters independently of immunity. Growth curves of a) KYSE150, MCF7, mEC25, and 4T1 cells upon MNX1 depletion or b) KYSE30 cells upon MNX1 overexpression, as determined by CCK‐8 assays. *n* = 6. c–e) Tumor growth of MNX1‐KO KYSE150 cells and control cells in nude mice. The images of established tumors (c) as well as the quantification of tumor weight (d) and volume (e) are shown (*n* = 10 per group). f–h) Tumor growth of MNX1‐overexpressing KYSE30 cells and control cells in nude mice. The images of established tumors (f) as well as the quantification of tumor weight (g) and volume (h) are shown (*n* = 9 per group). i,j) Migration and invasion abilities of ESCC cells upon MNX1 knockdown or overexpression, as determined by Transwell migration and invasion assays. Representative images (top) and quantitative analyses (bottom) are shown. *n* = 3. k) The expression of cell cycle‐related genes in KYSE150 cells upon MNX1 knockout, as determined by western blotting. The data are presented as mean ± SD, except for (e) and (h) expressed as mean ± SEM. The *p* value was calculated by two‐tailed unpaired Student's *t*‐test in (d,g,j), by one‐way ANOVA in (i), and by two‐way ANOVA in (a,b,e,h). *p < 0.05; **p < 0.01; ***p < 0.001.

Given that MNX1 depletion also inhibited the growth of 4T1 cells in immunodeficient mice, although the effect was weaker than that in immunocompetent mice, we thought that MNX1 may facilitate tumor progression independently of the immune system. Indeed, previous studies have reported that MNX1 functions as an oncogene in multiple cancers.^[^ ^30^, ^31^, ^32^, ^33^, ^34^, ^36^, ^37^ ^]^ However, the oncogenic effect independent of immune system in cancers, especially ESCC and breast cancer, is still unclear. Thus, we investigated the roles of MNX1 in cancer cells cultured in vitro and implanted in nude mice. As expected, Cell Counting Kit‐8 (CCK‐8) assays showed that the depletion of MNX1 suppressed the growth of human and mouse ESCC cells as well as human and mouse breast cancer cells in vitro (**Figure** **5**a and Figure S5a–c in Supporting Information). On the other hand, the ectopic expression of MNX1 promoted the growth of ESCC cells (Figure 5b). Colony formation assays in MNX1‐depleted or overexpressing ESCC cells further confirmed this result (Figure S5d,e in Supporting Information). Consistent with the effect of MNX1 in vitro, we found that depletion of MNX1 suppressed, while ectopic expression of MNX1 promoted the tumor growth of ESCC cells in vivo (Figure 5c–h). Furthermore, depletion of MNX1 inhibited the migration and invasion abilities of ESCC cells (Figure 5i and Figure S5f in Supporting Information), while ectopic expression of MNX1 showed the opposite trend (Figure 5j). Moreover, we examined the expression of some cell cycle and motility‐related genes and found that depletion of MNX1 could change their expression (Figure 5k and Figure S5g in Supporting Information). Therefore, MNX1 did not only participate in the anti‐tumor immunity, but might also promote tumor progression by regulating the cell cycle and motility, indicating that MNX1 contributes tumor progression in both immune‐dependent and independent manners, further supporting its pivotal role during carcinogenesis.

### 2.6 MNX1 Is Highly Expressed in ESCC and Is Located in Both Nucleus and Cytoplasm in Cancer Cells

**Figure 6:**
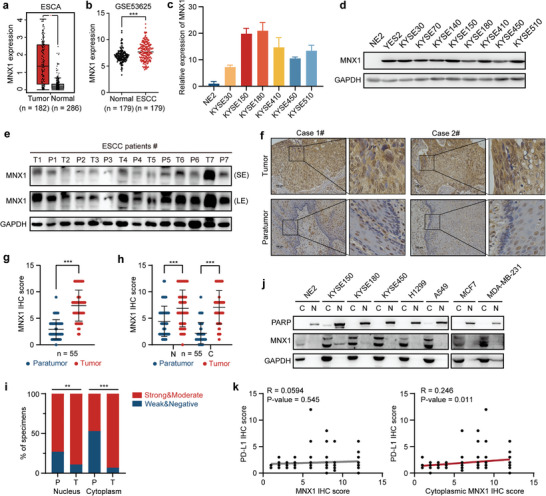
MNX1 is highly expressed in ESCC and is located in both nucleus and cytoplasm in cancer cells. a) The mRNA levels of MNX1 in ESCA tissues (*n* = 182) and normal tissues (*n* = 286), obtained from GEPIA database. b) The mRNA levels of MNX1 in ESCC tissues and normal tissues, based on GSE53625 dataset (*n* = 179). c) The mRNA and d) protein levels of MNX1 in immortalized esophageal epithelium cell line NE2 and ESCC cell lines, as determined by RT‐qPCR and western blotting. *n* = 3. e) The protein levels of MNX1 in 7 pairs of ESCC tumor tissues (T) and para‐tumor tissues (P), as detected by western blotting. SE: short‐time exposure; LE: long‐time exposure. f,g) The protein levels of MNX1 in 55 pairs of ESCC tumor tissues and para‐tumor tissues, as detected by IHC staining. Representative images (f) and quantitative analyses (g) are shown. h) The nuclear and cytoplasmic MNX1 levels in 55 pairs of ESCC tumor tissues and para‐tumor tissues, according to IHC staining. N, nucleus; C, cytoplasm. i) The percentage of MNX1 staining intensity in nucleus and cytoplasm of 55 pairs of ESCC tumor tissues (T) and para‐tumor tissues (P). j) The nuclear and cytoplasmic MNX1 levels in immortalized esophageal epithelium cell line NE2, ESCC cell lines (KYSE150, KYSE180, and KYSE450), lung cancer cell lines (H1299 and A549), as well as breast cancer cell lines (MCF7 and MDA‐MB‐231), as determined by western blotting following subcellular fractionation assay. GAPDH was used as the cytoplasmic protein control and PARP was served as the nuclear protein control. N, nucleus; C, cytoplasm. k) The correlation of PD‐L1 expression with total or cytoplasmic MNX1 in ESCC tumor tissues, based on IHC staining. *n* = 106. The data are presented as mean ± SD. The *p* value was calculated by two‐tailed paired Student's *t*‐test in (b,g,h), by Chi‐squared test in (i), and by Spearman's rank correlation coefficient in (k). **p < 0.01; ***p < 0.001.

Based on the critical role of MNX1 in promoting cancer progression, we next focused on the expression of MNX1 in human cancers. MNX1 has been reported to be highly expressed in multiple types of human cancers, such as LUAD,^[^ ^30^ ^]^ bladder cancer,^[^ ^33^ ^]^ breast cancer,^[^ ^35^ ^]^ and CRC.^[^ ^36^, ^37^ ^]^ However, the expression of MNX1 in ESCC is still unclear. By analyzing the Gene Expression Profiling Interactive Analysis (GEPIA) database (http://gepia.cancer‐pku.cn/)^[^ ^43^ ^]^ and GSE53625 dataset^[^ ^44^ ^]^ from the NCBI's Gene Expression Omnibus (GEO) database, we found that MNX1 mRNA was upregulated in ESCA as well as its subtype ESCC (**Figure** **6**a,b). To verify the high MNX1 expression in ESCC, we performed RT‐qPCR and western blotting assays, and observed that the expression of MNX1 in most ESCC cell lines and tissues was higher than that in immortalized esophageal epithelium cell line NE2 and paired para‐tumor tissues, respectively (Figure 6c–e). Consistently, immunohistochemistry (IHC) assay using ESCC microarray further showed that MNX1 was overexpressed in ESCC tissues (Figure 6f,g).

Interestingly, results from IHC staining showed that MNX1 was predominantly located in the nucleus in para‐tumor tissues, but it was also distributed in the cytoplasm in ESCC tissues (Figure 6f). Both cytoplasmic MNX1 and nuclear MNX1 were significantly upregulated in ESCC tissues compared with paired para‐tumor tissues (Figure 6h,i). In keeping with this result, subcellular fractionation assay revealed that a significant portion of MNX1 could be detected in the cytoplasm in several human cancer cells (Figure 6j). These results indicate that MNX1 exhibits a cytoplasmic distribution characteristic in cancer cells, supporting its role in regulating PD‐L1 mRNA stability in the cytoplasm. Then, we analyzed the correlation between MNX1 and PD‐L1 expression based on the IHC staining and found that cytoplasmic MNX1, but not total MNX1, was positively associated with PD‐L1 expression in cytoplasm and membrane (Figure 6k). Collectively, all of above results suggest that MNX1 is highly expressed in ESCC, especially in the cytoplasm, thus stabilizing PD‐L1 mRNA.

### 2.7 MNX1 Is Upregulated by Its Adjacent LncRNA MNX1‐AS1

**Figure 7:**
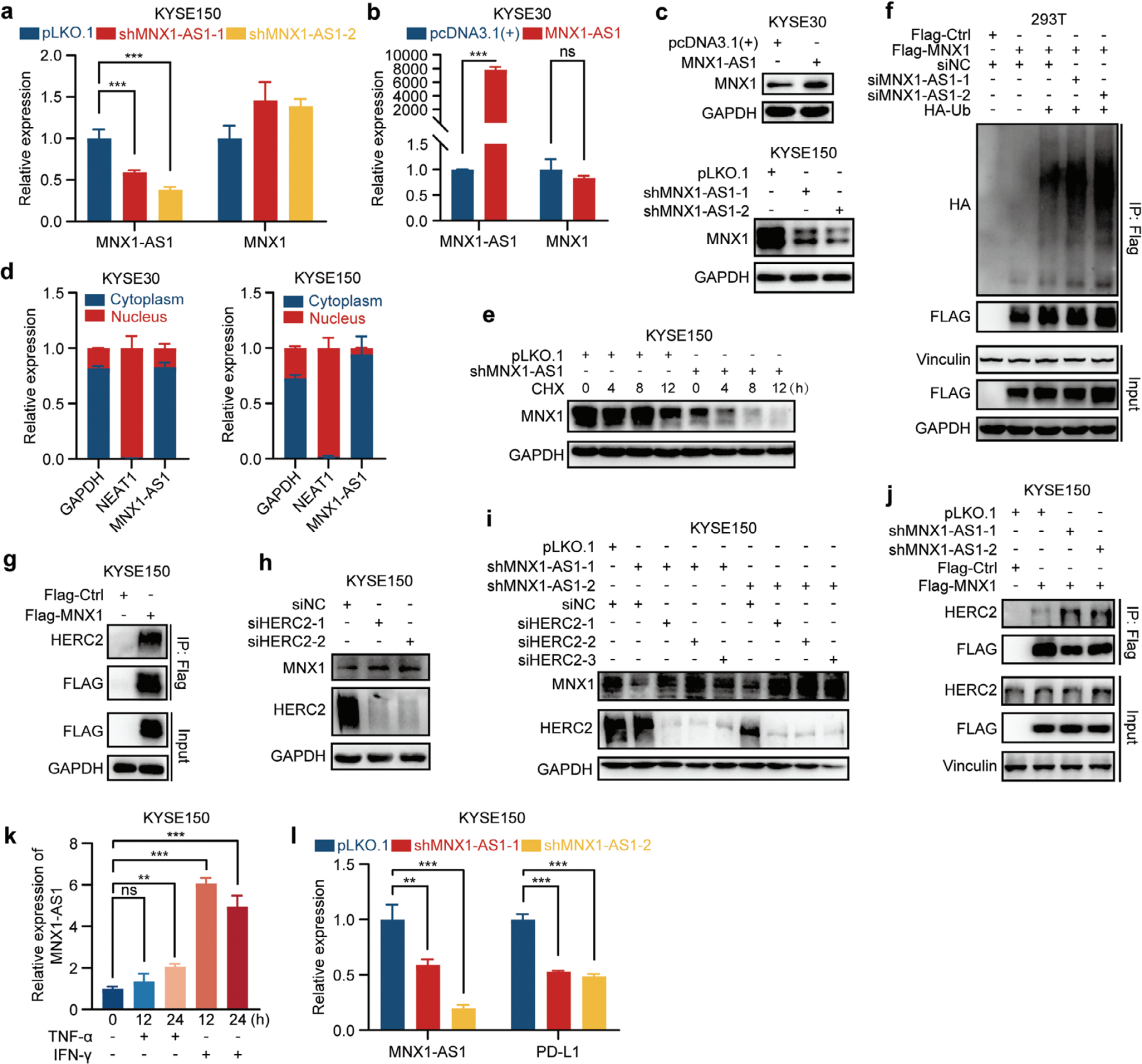
MNX1 is upregulated by its adjacent lncRNA MNX1‐AS1. a–c) The expression of MNX1‐AS1 and MNX1 in KYSE150 cells with depleted MNX1‐AS1 or KYSE30 cells transfected with pcDNA3.1(+)‐MNX1‐AS1 vector, as determined by RT‐qPCR and western blotting. d) The nuclear and cytoplasmic MNX1‐AS1 levels in ESCC cells, as detected by RT‐qPCR following subcellular fractionation assay. GAPDH was served as the cytoplasmic RNA control, while NEAT1 was used as the nuclear RNA control. e) The protein levels of MNX1 in MNX1‐AS1‐depleted KYSE150 cells treated with cycloheximide (CHX; 50 µg mL−1) for the indicated time points, detected by western blotting. f) IP coupled with western blotting analysis of MNX1 ubiquitination in HEK293T cells cotransfected with MNX1‐AS1 siRNA, Flag‐tagged MNX1 vector, and HA‐Ub (hemagglutinin‐tagged ubiquitin) vector for 24 h and then treated with bortezomib for 8 h before harvesting. g) IP coupled with western blotting showing the interaction between Flag‐tagged MNX1 and HERC2 in KYSE150 cells transfected with Flag‐tagged MNX1 vector for 24 h. h) The protein levels of MNX1 and HERC2 in KYSE150 cells transfected with HERC2 siRNAs or negative control siRNA, as determined by western blotting. i) The protein levels of MNX1 and HERC2 in MNX1‐AS1‐depleted KYSE150 cells transfected with HERC2 siRNAs, as determined by western blotting. j) IP coupled with western blotting showing the interaction between Flag‐tagged MNX1 and HERC2 in MNX1‐AS1‐depleted KYSE150 cells transfected with Flag‐tagged MNX1 vector for 24 h. k) The expression of MNX1‐AS1 in KYSE150 cells under the stimulation of TNF‐α (20 ng mL^−1^) or IFN‐γ (20 ng mL^−1^) for 12 and 24 h, as determined by RT‐qPCR. l) The expression of MNX1‐AS1 and PD‐L1 in MNX1‐AS1‐depleted KYSE150 cells and control cells, as determined by RT‐qPCR. The data are presented as mean ± SD (*n* = 3). The *p* value was calculated by two‐tailed unpaired Student's *t*‐test in (b) and by one‐way ANOVA in (a,k,l). **p < 0.01; ***p < 0.001; ns, no significant.

MNX1‐AS1 is a long non‐coding RNA (lncRNA) that transcribes in the opposite direction to MNX1 and has no overlapping exons with MNX1 (Figure S6a in Supporting Information). Increasing evidence has shown that lncRNA can regulate the expression of neighboring or host genes in *cis* or in *trans*.^[^ ^45^, ^46^ ^]^ MNX1‐AS1 has been reported to be overexpressed in multiple human cancers and facilitates tumor progression.^[^ ^47^, ^48^, ^49^ ^]^ Thus, we speculated that MNX1‐AS1 may contribute to the high expression of MNX1 in human cancers. Analysis of GEPIA database (http://gepia.cancer‐pku.cn/)^[^ ^43^ ^]^ showed that the expression of MNX1 was positively related to MNX1‐AS1 expression in human cancers, including ESCA and BRCA (Figure S6b in Supporting Information). However, knockdown or ectopic expression of MNX1‐AS1 did not correspondingly decrease or increase MNX1 mRNA level in ESCC cells (**Figure** **7**a,b), but altered MNX1 expression at the protein level (Figure 7c). Subcellular fractionation assay revealed that MNX1‐AS1 was predominantly enriched in the cytoplasm in ESCC cells (Figure 7d). Because MNX1 could also be localized in the cytoplasm in cancer cells, we then examined whether MNX1‐AS1 could regulate MNX1 protein stability. We used cycloheximide (CHX) to block protein synthesis and found that knockdown of MNX1‐AS1 accelerated the degradation of MNX1 protein in KYSE150 cells (Figure 7e). Moreover, in the presence of proteasome inhibitor bortezomib, knockdown of MNX1‐AS1 promoted the ubiquitination of MNX1 in HEK293T cells (Figure 7f and Figure S6c in Supporting Information), collectively indicating that MNX1‐AS1 increases MNX1 protein level via inhibiting its degradation.

We then asked which E3 ubiquitin ligase is involved in the degradation of MNX1. By analyzing the mass spectrometry results of differential bands as described above (Figure 3d,e), we found that there was no E3 ubiquitin ligase in these candidate MNX1‐interacting proteins. To comprehensively search for the binding proteins of MNX1, we conducted mass spectrometry analysis on whole bands and discovered an E3 ubiquitin ligase, HECT and RLD domain containing E3 ubiquitin protein ligase 2 (HERC2),^[^ ^50^, ^51^ ^]^ which was also predicted by UbiBrowser (http://ubibrowser.bio‐it.cn/ubibrowser/)^[^ ^52^ ^]^ as one of the E3 ubiquitin ligases of MNX1 (Figure S6d in Supporting Information). IP assay combined with western blotting confirmed that MNX1 was able to interact with HERC2 (Figure 7g). Expectedly, siRNA‐mediated depletion of HERC2 led to an increase in MNX1 protein level in KYSE150 cells (Figure 7h), suggesting that HERC2 can regulate the expression of MNX1. Moreover, the depletion of HERC2 could rescue MNX1 expression downregulated by MNX1‐AS1 knockdown (Figure 7i), indicating that HERC2 is involved in the MNX1‐AS1‐mediated regulation of MNX1 expression. Furthermore, IP assay revealed that depletion of MNX1‐AS1 in KYSE150 cells increased the interaction between HERC2 and MNX1 (Figure 7j). In addition, we observed that MNX1‐AS1 could also respond to the inflammatory cytokines IFN‐γ and TNF‐α (Figure 7k). And, the knockdown of MNX1‐AS1 resulted in a decrease in PD‐L1 mRNA level (Figure 7l). These data collectively indicate that MNX1‐AS1 enhances MNX1 expression in ESCC cells by stabilizing its protein.

### 2.8 Depletion of MNX1 Sensitizes CTLA‐4 Blockade Therapy

**Figure 8:**
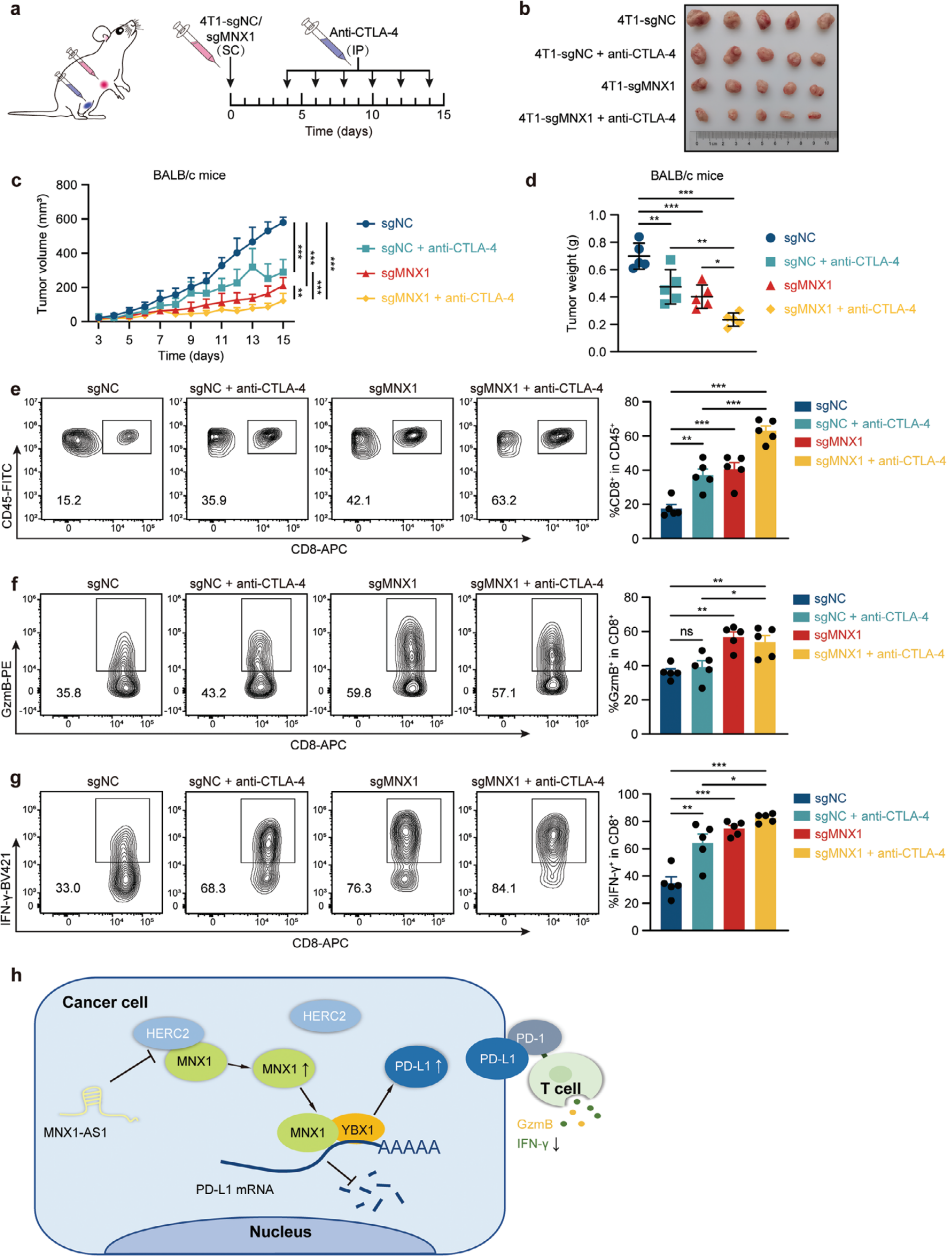
Depletion of MNX1 sensitizes CTLA‐4 blockade therapy. a) Schematic diagram of anti‐CTLA‐4 antibody dosage regimen in BALB/c mouse tumor model. Mice bearing tumors of MNX1‐KO 4T1 cells or control cells were treated with anti‐CTLA‐4 antibody as indicated. SC, subcutaneous injection; IP, intraperitoneal injection. b–d) Tumor growth of MNX1‐KO 4T1 cells or control cells in anti‐CTLA‐4 antibody‐treated BALB/c mice. The images of established tumors (b) as well as the quantification of tumor volume (c) and weight (d) are shown (*n* = 5 per group). e) CD8^+^ T cell populations in tumors of anti‐CTLA‐4 antibody‐treated BALB/c mice as well as f) GzmB‐expressing and g) IFN‐γ‐expressing CD8^+^ T cells were analyzed by flow cytometry. Representative images (left) and quantitative analyses (right) are shown (*n* = 5 per group). h) Schematic diagram of MNX1 promoting cancer progression through PD‐L1‐mediated immune evasion. The data are presented as mean ± SD, except for (e–g) expressed as mean ± SEM. The *p* value was calculated by two‐way ANOVA in (c) and by one‐way ANOVA in (d–g). *p < 0.05; **p < 0.01; ***p < 0.001; ns, no significant.

Finally, we aimed to explore whether MNX1 ablation can improve the efficacy of ICB‐based immunotherapy. Immunosuppressive checkpoints, such as PD‐L1 and CTLA‐4, can help cancer cells evade immune surveillance via different mechanisms. Combination of anti‐PD‐1 with anti‐CTLA‐4 has been reported to have a better therapeutic efficacy.^[^ ^53^ ^]^ Therefore, based on the decreased PD‐L1 levels after MNX1 depletion, we evaluated the impact of MNX1 ablation on anti‐CTLA‐4 therapy. To do it, we inoculated MNX1‐KO 4T1 cells and control cells into BALB/c mice (immune competent), respectively, and then treated the mice with anti‐CTLA‐4 antibody (**Figure** **8**a). Expectedly, anti‐CTLA‐4 therapy suppressed the tumor growth of 4T1 cells in vivo (Figure 8b–d). Importantly, the depletion of MNX1 further enhanced the therapeutic efficacy of anti‐CTLA‐4 antibody (Figure 8b–d). Flow cytometry analysis revealed that anti‐CTLA‐4 therapy combined with MNX1 depletion increased the CD8^+^ T cell infiltration in tumors as compared to the blockade of CTLA‐4 alone (Figure 8e). The combination also increased the production of GzmB and IFN‐γ among CD8^+^ T cells (Figure 8f,g). Altogether, our data suggest that depletion of MNX1 can enhance the therapeutic efficacy of CTLA‐4 blockade.

## 3 Discussion

Immunotherapy using PD‐L1/PD‐1 blockade can re‐activate the immune system to attack cancer cells, which has revolutionarily changed the cancer therapy.^[^ ^10^, ^54^ ^]^ PD‐L1 expression is associated with the response to PD‐1 or PD‐L1 inhibitors.^[^ ^11^, ^12^ ^]^ Thus, it is urgent to further reveal the underling mechanisms regulating PD‐L1 expression. In this study, we found that MNX1 can increase the stability of PD‐L1 mRNA, which is a novel function of MNX1 independent of transcriptional activity. Mechanistically, MNX1 interacts with YBX1 and enhances the binding of YBX1 to PD‐L1 mRNA. Depletion of MNX1 stimulates T cell anti‐tumor immunity, inhibits tumor progression, and sensitizes CTLA‐4 blockade therapy. MNX1 is upregulated in ESCC, and its adjacent lncRNA MNX1‐AS1 contributes to the upregulation at protein level in an E3 ubiquitin ligase HERC2‐dependent manner. Therefore, our study reveals a novel mechanism for regulating PD‐L1 expression via MNX1‐mediated PD‐L1 mRNA stabilization, and identified MNX1 as a potential target for tumor immunotherapy.

MNX1 has been reported to promote the progression of human cancers, however whether MNX1 can regulate anti‐tumor immunity is still unclear. Here, by using an online database and analyzing RNA‐seq data of MNX1‐depleted ESCC cells, we found that MNX1 was closely associated with cancer immunity. Moreover, the inflammatory cytokines IFN‐γ and TNF‐α were capable of inducing MNX1 expression in ESCC cells. Importantly, the depletion of MNX1 downregulated, while the ectopic expression of MNX1 upregulated PD‐L1 expression at both mRNA and protein levels in multiple cancer cell lines, which was due to stabilization of PD‐L1 mRNA rather than activation of transcription in ESCC cells.

MNX1 is a homeobox domain‐containing transcription factor, which has been reported to be located in the nucleus^[^ ^55^ ^]^ and transcriptionally regulate gene expression in human cancer cells.^[^ ^30^, ^33^, ^34^ ^]^ In our study, IHC staining showed that MNX1 was indeed predominantly located in the nucleus in para‐tumor tissues, but in ESCC tissues, MNX1 was localized in both cytoplasm and nucleus, and cytoplasmic MNX1 was positively associated with PD‐L1 expression. Likewise, subcellular fractionation assay revealed that MNX1 was obviously expressed in the cytoplasm in several cancer cell lines. IP assay, mass spectrometry analysis, and western blotting validation showed that MNX1 was capable of interacting with YBX1, a DNA/RNA‐binding protein involved in numerous DNA/RNA‐dependent events, including mRNA stability.^[^ ^41^, ^42^ ^]^ Moreover, we found that YBX1 was able to bind to the 3`UTR of PD‐L1 mRNA, while depletion or overexpression of MNX1 inhibited or promoted this binding. Simultaneous depletion of MNX1 and YBX1 had no significant synergistic effect on the downregulation of PD‐L1. Furthermore, the stability of PD‐L1 mRNA induced by MNX1 overexpression was reduced upon YBX1 knockdown. These results indicate that MNX1 stabilizes PD‐L1 mRNA by interacting with YBX1 and promoting the binding of YBX1 to PD‐L1 mRNA.

Although previous studies have demonstrated that MNX1 facilitates the malignant phenotypes of cancer cells, such as proliferation, invasion, and migration,^[^ ^30^, ^31^, ^32^, ^33^, ^34^, ^36^, ^37^ ^]^ these experiments were performed in tumor cells cultured in vitro or implanted in immunodeficient mice. Here, we found that depletion of MNX1 exhibited a more significant tumor suppressive effect in immunocompetent mice than that in immunodeficient mice. Flow cytometry analysis of implanted tumors in immunocompetent mice showed that the depletion of MNX1 increased CD8^+^ T cell infiltration as well as IFN‐γ and GzmB production, indicating that MNX1 ablation activates T cell anti‐tumor immunity, thereby further inhibiting tumor growth. Importantly, compared to using only one regimen, combination of MNX1 depletion with anti‐CTLA‐4 antibody significantly inhibited the tumor growth, indicating that targeting MNX1 may be a potential method to enhance the therapeutic efficacy of CTLA‐4 inhibitor.

Database analysis or clinical tissue verification have shown that MNX1 is upregulated in multiple human cancers, such as breast cancer and LUAD, and increased expression of MNX1 is associated with poor prognosis.^[^ ^30^, ^31^, ^33^, ^34^, ^35^, ^36^, ^37^ ^]^ Here, we demonstrated that the expression of MNX1 was also elevated in ESCC. We found that lncRNA MNX1‐AS1, which transcribes from the opposite strand to MNX1, increased MNX1 expression at protein level rather than mRNA level by regulation of MNX1 protein stability in ESCC cells.

In summary, we explored a novel immunosuppressive mechanism regulated by MNX1 in cancer cells. We proposed a model (Figure 8h), in which MNX1 protein, stabilized by lncRNA MNX1‐AS1, enhances PD‐L1 mRNA stability by binding to YBX1, thus leading to increased PD‐L1 abundance and subsequent immune escape. Our study reveals a novel function of MNX1 in promoting tumor growth through PD‐L1‐mediated immune evasion. Therefore, MNX1 might be a promising therapeutic target for cancer patients.

## 4 Experimental Section

### Cell Culture and Treatments

Human ESCC cell lines (YES2, KYSE30, KYSE70, KYSE140, KYSE150, KYSE180, KYSE410, KYSE450, and KYSE510) were kindly provided by Dr. Yutaka Shimada (Kyoto University, Japan), immortalized esophageal epithelium cell line NE2 was kindly provided by Prof. Enmin Li (Shantou University, China), and mouse ESCC cell line mEC25 was kindly provided by Prof. Li Fu (Shenzhen University, China). Human breast cancer cell lines (MCF7 and MDA‐MB‐231) and mouse breast cancer cell line 4T1, human lung cancer cell lines (NCI‐H1299 and A549), as well as HEK293T and Jurkat cell lines were obtained from the American Type Culture Collection (ATCC). Jurkat, 4T1, NCI‐H1299, and human ESCC cell lines were maintained in RPMI 1640 medium (Gibco). HEK293T, MCF7, and mEC25 cells were maintained in Dulbecco's modified Eagle medium (DMEM; Gibco). A549 cells were cultured in Ham's F‐12K medium (Gibco), while MDA‐MB‐231 cells were cultured with Leibovitz's L‐15 medium (Gibco). All medium was supplemented with 10% fetal bovine serum (FBS) and 1% penicillin/streptomycin, and all cells except MDA‐MB‐231 were cultured in an incubator with 5% CO_2_ at 37 °C. To inhibit the transcription, exponentially growing cancer cells were treated with 5 µg mL^−1^ of actinomycin D (ACTD; APExBIO, USA) for the indicated times. To inhibit the translation, exponentially growing cancer cells were treated with 50 µg mL^−1^ of cycloheximide (CHX; Selleck, USA) for 4, 8, or 12 h. For inflammatory cytokine (PeproTech, USA) treatments, exponentially growing cancer cells were treated with 20 ng mL^−1^ of IFN‐γ or TNF‐α for the indicated times. To inhibit the proteasome, HEK293T cells were treated with 20 nm of bortezomib (Selleck, USA) for 8 h.

### Clinical Samples and Ethics

The fresh ESCC tissues and para‐tumor tissues were collected from the primary ESCC patients, who underwent surgery at the Fourth Hospital of Hebei Medical University. All patients did not receive preoperative radiotherapy and chemotherapy. The informed consent was provided by patients and the study was approved by the Ethics Committee of the Fourth Hospital of Hebei Medical University (approval no. 2021KY080).

### Gene Knockdown and Knockout

For stable knockdown, shRNAs targeting MNX1 or MNX1‐AS1 were synthesized, annealed, and cloned into pLKO.1 plasmids. The pLKO.1‐MNX1‐shRNA plasmids or pLKO.1‐MNX1‐AS1‐shRNA plasmids, together with lentiviral packaging plasmids pMD2.G and psPAX2, were introduced into HEK293T cells using Neofect DNA transfection reagent (Neofect (beijing) Biotech, China) per manufacturer's protocol. 48–72 h later, the medium containing lentiviral particles was collected by centrifugation at 1000 rpm for 5 min and filtration with 0.45 µm syringe filter. The lentiviral particles were then used to infect ESCC or breast cancer cells for 24 h in the presence of 8 µg mL^−1^ polybrene (Beyotime Biotechnology, China). Lentiviruses derived from pLKO.1 empty plasmids were used as the negative control. After selection with 2 µg mL^−1^ of puromycin (Solarbio, China) for 5 d, the stably MNX1 or MNX1‐AS1‐depleted cell lines were obtained and maintained in the medium containing 1 µg mL^−1^ of puromycin.

For transient knockdown, siRNAs targeting MNX1, MNX1‐AS1, HERC2, and YBX1, purchased from Guangzhou RiboBio Co., Ltd. (China), were introduced into cells using Lipofectamine 2000 transfection reagent (Invitrogen, USA) according to the manufacturer's instructions.

For CRISPR/Cas9‐mediated MNX1 knockout, single‐guide RNA (sgRNA) targeting MNX1 (sgMNX1) was cloned into lentiCRISPRv2 plasmids by Beijing Tsingke Biotech Co., Ltd. (China). The lentiviral particles carrying sgMNX1 were packaged and used to infect ESCC or breast cancer cells. MNX1‐KO monoclonal cell lines were generated by diluting cells in 96‐well plates. The target sequences of shRNAs, siRNAs, and sgRNA are listed in Table S1 (Supporting Information).

### Gene Overexpression

The coding sequence (CDS) of human MNX1 was cloned into lentiviral vector pCDH‐CMV‐MCS‐EF1‐CopGFP‐T2A‐Puro with a C‐terminal Flag tag by WZ Biosciences Inc. (Shandong, China). Lentiviruses containing MNX1 CDS were packaged and used to infect KYSE30 cells to generate stably MNX1‐overexpressing KYSE30 cell line. For overexpression of MNX1‐AS1, the cDNA of MNX1‐AS1 was cloned into pcDNA3.1(+) plasmids. KYSE30 cells were transfected with pcDNA3.1(+)‐MNX1‐AS1 plasmids or pcDNA3.1(+) empty plasmids using Neofect DNA transfection reagent (Neofect (beijing) Biotech, China) according to the manufacturer's protocol.

### Cell Growth and Colony Formation Assays

Cell viability was measured by CCK‐8 reagent (APExBIO, USA) according to the manufacturer's instructions. Briefly, cells were seeded in 96‐well plates with complete medium at a density of 1 × 10^3^ or 2 × 10^3^ cells per well. 10 µL of CCK‐8 solution was added to each well every day and incubated for 2 h at 37 °C. The absorbance was detected at 450 nm by a microplate reader (Sunrise, TECAN). For colony formation assay, cells in single‐cell suspension were planted into six‐well plates or dishes. After about 2 weeks, the colonies were fixed with methanol and stained with 1% crystal violet solution. After being washed and completely dried, the colonies were photographed and counted.

### Transwell Migration and Invasion Assays

Migration assay was performed in a 24‐well Transwell plate with 8.0 µm pore polycarbonate membrane inserts (Corning). 600 µL of 20% FBS‐containing medium was added to the lower chamber, and 3 × 10^5^ cells (KYSE150 or KYSE410) or 2 × 10^5^ cells (KYSE30) in 100 µL of FBS‐free medium were planted into the upper chamber and incubated at 37 °C overnight. The chambers were fixed with methanol and stained with 1% crystal violet solution. After remove of the non‐migrating cells on the inside membrane, cells on the outside membrane were photographed and counted. Invasion assay was conducted as described in migration assay except that the membrane was pre‐coated with Matrigel matrix.

### Tumor‐T Cell Co‐Culture Assay

Jurkat‐mCherry cells (expressing mCherry fluorescent protein) were activated in the presence of CD3 antibody (1 µg mL^−1^; BioLegend) and CD28 antibody (1 µg mL^−1^; BioLegend) for 24 h. The activated Jurkat cells were then added to MNX1‐KO KYSE150 cells or control cells at a ratio of 10:1. Three days later, the co‐cultured cells were analyzed by flow cytometry.

### RNA Isolation and RT‐qPCR

Total RNA of cells or RIP samples was extracted using RNAiso Plus (Takara) according to the standard protocols. cDNA was generated with HiScript II Q RT SuperMix for qPCR (+gDNA wiper) (Vazyme Biotech Co., Ltd., China). qPCR reactions were carried out using MonAmp ChemoHS qPCR Mix (Monad, China) on QuantStudio3 Real‐Time PCR System (Applied Biosystems, USA) or LineGene 9600 Plus Real‐Time PCR System (Bioer Technology, China). The expression levels of target genes were calculated using the 2^−ΔΔCT^ method and normalized to GAPDH. Primers used for qPCR are listed in Table S2 (Supporting Information).

### Western Blotting

Cells and frozen tissues were lysed by using RIPA lysis buffer (Proteintech, China) supplemented with phosphatase inhibitor and protease inhibitor. Cell debris was removed by centrifugation at 12 000 rpm for 20 min at 4 °C. Protein concentration in supernatant fractions was detected using BCA Protein Assay Kit (Beyotime Biotechnology, China). An equal amount of protein sample was run on SDS‐PAGE and transferred onto nitrocellulose membranes (Millipore, USA). After blocked with 5% non‐fat milk, the membranes were incubated with primary antibodies at 4 °C overnight. Antibodies used are as follows: anti‐Cyclin D3 (Cell Signaling Technology, #2936S, 1:1000), anti‐Cyclin E1 (Cell Signaling Technology, #4129, 1:1000), anti‐CDK2 (Cell Signaling Technology, #2546S, 1:1000), anti‐CDK6 (Cell Signaling Technology, #3136, 1:1000), anti‐p27 (Cell Signaling Technology, #3686S, 1:1000), anti‐PD‐L1 (Cell Signaling Technology, #13684, 1:1000), anti‐MNX1 (Immunoway, YN0875, 1:1000), anti‐PARP (Cell Signaling Technology, #9542S, 1:1000), anti‐HA (Proteintech, 81290‐1‐RR, 1:5000), anti‐HERC2 (Proteintech, 27459‐1‐AP, 1:1000), anti‐YBX1 (Proteintech, 20339‐1‐AP, 1:5000), anti‐Flag (Sigma‐Aldrich, F3165, 1:1000), anti‐Vinculin (Proteintech, 66305‐1‐Ig, 1:5000), and anti‐GAPDH (Proteintech, 60004‐1‐Ig, 1:5000). The next day, membranes were washed three times and incubated with secondary antibody at room temperature for 1 h. Blots were imaged using ECL Western Blotting Substrate (Tanon, China) on MiniChemi Chemiluminescent Imaging System (SAGECREATION, China).

### RNA‐seq and Analyses

Total RNA of KYSE150 cells transfected with MNX1 siRNA or negative control siRNA was isolated using RNAiso Plus (Takara). Then, library preparation, reads sequencing, and analysis were performed by Novogene Bioinformatics Technology Co., Ltd. (China). Briefly, mRNA, enriched from the total RNA using poly‐T oligo‐attached magnetic beads, was used to construct the library. Sequencing was then performed on Illumina NovaSeq 6000. Gene expression levels were estimated by fragments per kilobase of transcript per million mapped reads (FPKM). Differential expression genes (DEGs) were determined with *p* value < 0.05 and |log2(foldchange)| ≥ 1 as a threshold. GO enrichment analysis of the DEGs was performed in DAVID website.^[^ ^56^, ^57^ ^]^

### Subcellular Fractionation Assay

For fractionation of nuclear and cytoplasmic proteins, the subcellular fractionation was carried out using Nuclear and Cytoplasmic Protein Extraction Kit (Beyotime Biotechnology, China) according to the Kit's instructions. Then, the extracted nuclear and cytoplasmic proteins were quantified with BCA Protein Assay Kit (Beyotime Biotechnology, China) and the levels of MNX1 were examined by western blotting. PARP was used as the nuclear protein reference, while GAPDH was served as the cytoplasmic protein control.

For fractionation of nuclear and cytoplasmic RNAs, cells were suspended in hypotonic buffer (25 mm Tris‐HCl (pH = 7.4), 1 mm MgCl_2_, and 5 mm KCI) and incubated on ice for 10 min; then, an equal volume of 1% NP‐40‐containing hypotonic buffer was added and incubated for 10 min again, as described previously with some modifications.^[^ ^58^ ^]^ After centrifugation at 1000 *g* for 5 min at 4 °C, the supernatant was collected as cytoplasmic fraction and RNAiso Plus (Takara) was added to extract cytoplasmic RNA. The pellets were washed with PBS twice and RNAiso Plus was added to extract nuclear RNA. NEAT1 was used as the nuclear RNA reference, while GAPDH was served as the cytoplasmic RNA control.

### Dual‐Luciferase Reporter Assay

pGL3‐PD‐L1‐promoter plasmid was a gift from Prof. Wei Guo (Dalian Medical University, China). MNX1‐depleted KYSE150 cells or control cells as well as HEK293T cells were seeded in 24‐well plates. The next day, MNX1‐depleted KYSE150 cells or control cells were cotransfected with pGL3‐PD‐L1‐promoter plasmids and pRL‐TK plasmids using Neofect DNA transfection reagent (Neofect (beijing) Biotech, China) per manufacturer's protocol. HEK293T cells were cotransfected with pGL3‐PD‐L1‐promoter plasmids, pRL‐TK plasmids, and MNX1 siRNA or negative control siRNA using Lipofectamine 2000 transfection reagent (Invitrogen, USA) according to the manufacturer's protocol. After 24 h of transfection, the luciferase activities were measured by Dual‐Luciferase Reporter Assay System (Promega Corporation, USA) on a multimode plate reader (PerkinElmer, USA) according to the manufacturers’ instructions. Relative firefly luciferase activity was normalized by Renilla luciferase activity.

### RNA Pull‐Down Assay

The linear DNA templates, for in vitro transcription of sense or antisense PD‐L1 mRNA fragments, were obtained through PCR reaction using primers containing T7 promoter sequence. The primers used for PCR are listed in Table S2 (Supporting Information). In vitro transcription was performed with T7 High Yield RNA Transcription Kit (Vazyme Biotech Co., Ltd., China) according to the Kit's instructions and transcriptional RNA was labelled with biotin‐16‐UTP (Invitrogen, USA).

For RNA pull‐down assay, streptavidin agarose beads (Thermo Fisher Scientific) were washed several times with binding buffer (10 mm HEPES pH 7.0, 50 mm KCl, 10% glycerol, 1 mm EDTA, 1 mm DTT, 0.5% Triton X‐100). KYSE150 cells were lysed with RIPA lysis buffer (Proteintech, China) added with protease inhibitor. To remove the non‐specific interacting proteins, cell extracts were pre‐incubated with beads for 15 min at room temperature. To restore RNA secondary structure, 5 pmol of biotin‐labelled RNA was added to RNA structure buffer (10 mm Tris‐HCl pH 7.0, 0.1 m KCl, 10 mm MgCl_2_), heated at 95 °C for 2 min, cooled on ice for 3 min, and finally placed at room temperature for 30 min.^[^ ^59^ ^]^ Pre‐treated cell extracts were then incubated with biotin‐labelled, folded RNA for 1 h at room temperature in binding buffer supplemented with protease inhibitor and RNase inhibitor. After that, pre‐washed streptavidin agarose beads were added to each sample and incubated for 30 min at room temperature. After washing the non‐specific binding proteins, the retrieved proteins were subjected to western blotting analysis.

### RIP Assay

RIP assay was performed by Magna RIP RNA‐Binding Protein Immunoprecipitation Kit (Millipore, USA) according to the manufacturer's instructions. Briefly, MNX1‐KO KYSE150 cells or control cells, as well as KYSE150 cells transfected with Flag‐tagged MNX1 vector or Flag empty vector for 24 h, were lysed in RIP lysis buffer containing RNase inhibitor and protease inhibitor cocktail. Magnetic beads were washed with RIP wash buffer and incubated with IgG or anti‐YBX1 antibody (Proteintech, 20339‐1‐AP) for 30 min at room temperature. Then, cell extracts were incubated with magnetic beads conjugated with antibody at 4 °C overnight. Immunoprecipitated RNA was extracted and reversely transcribed into cDNA for qPCR analysis.

### IP Assay and Mass Spectrometry Analysis

To identify the proteins interacting with MNX1, KYSE150 cells were transfected with Flag‐tagged MNX1 vector or Flag empty vector by using Neofect DNA transfection reagent (Neofect (beijing) Biotech, China) according to the manufacturer's protocol. After 24 h, the transfected cells were lysed by IP lysis buffer (50 mm Tris‐HCl pH 7.4, 150 mm NaCl, 1 mm EDTA, 1% Triton X‐100, PMSF, and Cocktail) for 30 min. Cell lysates were centrifuged at 12 000 rpm for 20 minutes at 4 °C. The supernatants were then incubated with anti‐Flag M2 Affinity Gel (Sigma‐Aldrich) at 4 °C overnight. After washing the beads with washing buffer (50 mm Tris‐HCl pH 7.4, 150 mm NaCl, 0.5% Triton X‐100, 10% Glycerol) for 6 times, the immunoprecipitated protein samples were subjected to SDS‐PAGE and silver staining. The differential or whole bands were identified by mass spectrometry analysis (Shanghai iProteome Biotechnology Co., Ltd., China) with Q Exactive HF‐X (Thermo Fisher Scientific). GO enrichment analysis of the differential proteins was performed in DAVID website.^[^ ^56^, ^57^ ^]^ To validate the mass spectrometry results, the immunoprecipitated proteins were subjected to western blotting with specific antibodies.

### IHC

The human ESCC tissue microarray with PD‐L1 IHC staining data was purchased from Shanghai Outdo Biotech Co., Ltd. (China). After deparaffinized with dimethylbenzene and rehydrated with a series of gradually decreasing concentrations of ethanol, the slide was treated with H_2_O_2_ to block the endogenous peroxidase and was heated in sodium citrate (pH 6.0) for antigen retrieval. Then, the slide was blocked by goat serum working solution (ZSGB‐BIO, China) for 20 min at room temperature and was then incubated with anti‐MNX1 antibody (1:200 in PBS; Abcam, ab92606) at 4 °C overnight. The next day, the slide was incubated with second antibody for 1 h at room temperature. MNX1 was visualized by DAB staining and cell nucleus was counterstained with hematoxylin. The slide was ultimately dehydrated and mounted.

The positive staining cells were classed as 0 (no positive cells), 1 (1%‐25%), 2 (26%–50%), 3 (51%‐75%), and 4 (75%‐100%). The staining intensity was graded as 0 (negative), 1 (weak staining), 2 (moderate staining), and 3 (strong staining). By multiplying these two parameters, a value reflecting the level of MNX1 expression was obtained.

### In Vivo Mouse Study

All animal experiments were conducted in accordance with the guidelines approved by the Institutional Animal Care and Use Committee of Dalian Medical University. Mice were purchased from Beijing Vital River Laboratory Animal Technology Co., Ltd. (China) or Liaoning Changsheng Biotechnology Co., Ltd. (China) and were housed in a specific pathogen‐free (SPF) animal room. All types of tumor cells were subcutaneously injected into randomly assigned mice.

For the xenograft tumor models, human MNX1‐overexpressing KYSE30 cells or control cells as well as MNX1‐KO KYSE150 cells or control cells (1 × 10^6^ cells in 100 µL PBS per mouse) were subcutaneously injected into six‐week‐old male NU/NU nude mice. For the syngeneic tumor models, murine MNX1‐KO 4T1 cells or control cells (2 × 10^5^ cells in 100 µL PBS per mouse) were subcutaneously injected into six‐week‐old male BALB/c mice or BALB/c nude mice. After 5 d post‐injection, the tumor volume was measured every 1–4 d using a digital caliper and calculated by the formula: width^2^ × length/2.

For the immunotherapy mouse model with anti‐CTLA‐4 antibody, murine MNX1‐KO 4T1 cells or control cells (2 × 10^5^ cells in 100 µL PBS per mouse) were injected into six‐week‐old male BALB/c mice. The anti‐CTLA‐4 antibody (200 µg per mouse; Bio X Cell, #BE0164) was intraperitoneally injected into the mice at 4, 6, 8, 10, 12, and 14 d after tumor inoculation. The tumor volume was measured every day using a digital caliper.

### Flow Cytometry

For cultured cell lines, cells were detached from the culture dish with 0.02% EDTA in PBS and were then stained with anti‐PD‐L1 antibody conjugated with PE fluorescence (eBioscience). For the co‐cultured Jurkat‐mCherry cells and cancer cells, the total cells were collected and stained with fluorescently labelled anti‐human IFN‐γ antibody with PE‐Cy7 (BioLegend) and anti‐human/mouse Granzyme B antibody with PE (BioLegend). For subcutaneous tumor models, excised tumors were dissociated to single‐cell suspensions with a Tumor Dissociation Kit (Miltenyi Biotec). Lymphocytes were isolated with Percoll gradient (40%/80%; Solarbio) and was activated using 2% Cell Activation Cocktail (with Brefeldin A) (BioLegend) for 4 h at 37 °C. Lymphocytes were stained with fluorescently labelled anti‐mouse CD8 antibody with APC (eBioscience), anti‐mouse CD45 antibody with FITC (BioLegend), anti‐mouse IFN‐γ antibody with PE‐Cy7 or BV421 (BioLegend), and anti‐human/mouse Granzyme B antibody with PE (BioLegend). Stained samples were analyzed on NovoCyte or Quanteon (Agilent) using NovoExpress software, and the data were analyzed with FlowJo software (v9.32, Tree Star).

### Statistics

Data are presented as mean ± SD unless otherwise specified in the figure legends. Statistical significance was determined by two‐tailed Student's *t*‐test, one‐way analysis of variance (ANOVA), or two‐way ANOVA, as indicated in the corresponding figure legends. The correlation between MNX1 and PD‐L1 expression based on IHC staining was examined by Spearman's rank correlation coefficient. All statistical analyses were performed using GraphPad Prism 9.5 software. Statistical significance was set as follows: *p < 0.05; **p < 0.01; ***p < 0.001; ns, not significant.

## Conflict of Interest

The authors declare no conflict of interest

## Supporting information



Supporting Information

Supporting Table 1

Supporting Table 2

## Data Availability

The data that support the findings of this study are available from the corresponding author upon reasonable request.
