# Supplementary material for: Loss of MNX1 Sensitizes Tumors to Cytotoxic T Cells by Degradation of PD‐L1 mRNA
Source: Adv Sci (Weinh). 2025 Feb 6;12(12):2403077. doi: 10.1002/advs.202403077 (PMC11947991; doi:10.1002/advs.202403077)
Supplement: Supplementary file 1 — Supporting Information [file ADVS-12-2403077-s002.docx]

**Supporting Information**

**Loss of MNX1 Sensitizes Tumors to Cytotoxic T Cells by Degradation of PD-L1 mRNA**

*Zhengzheng Li^#^, Lei Chen^#^, Ge Zhang^#^, Shuang Wang^#^, Enhang Xu, Jinglei Teng, Jiancheng Xu, Fang Peng, Qingjie Min, Zhuoya Wang, Shujuan Shao, Lianmei Zhao, Baoen Shan, Yang Wang, Qimin Zhan^*^ and Xuefeng Liu^*^*

#Contributed equally to this work

^*^Correspondence

**Figure S1**


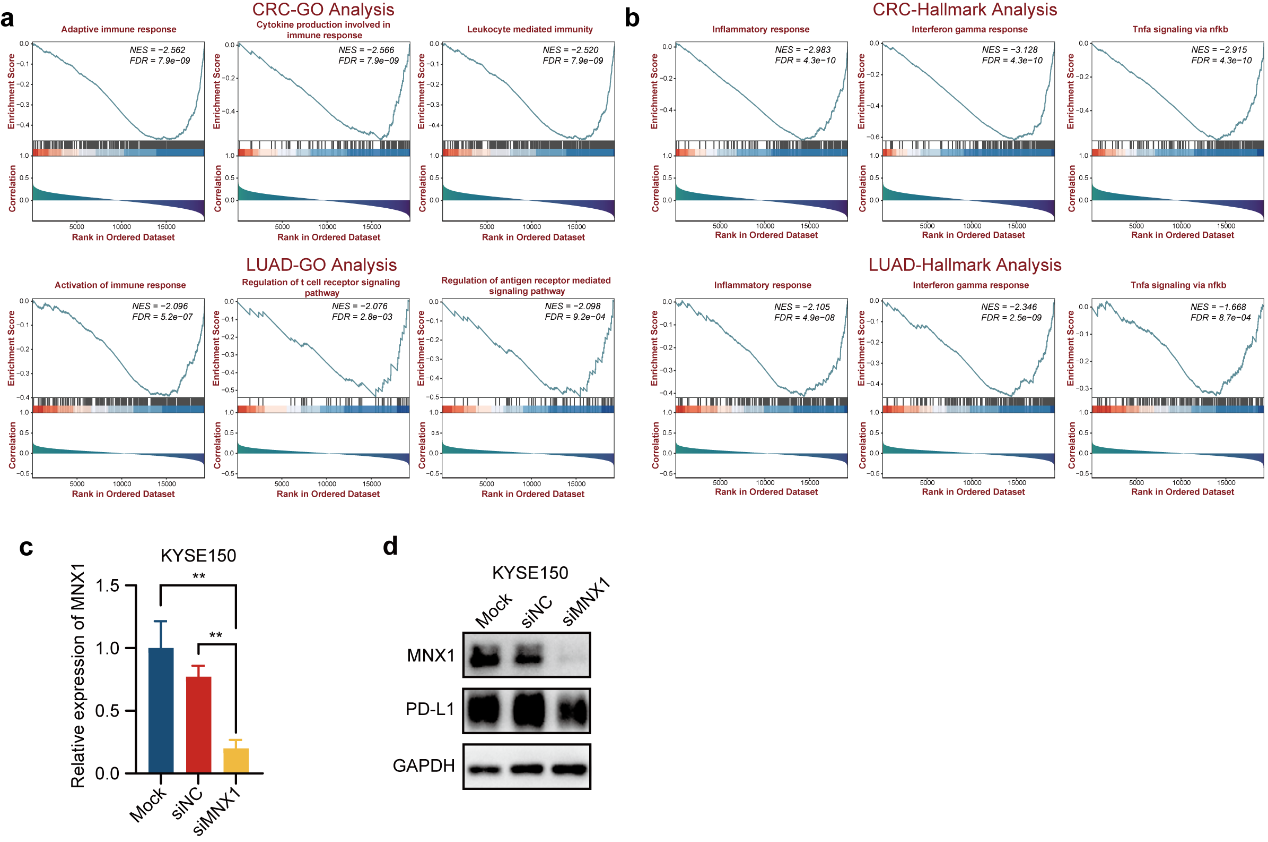


a, b) GSEA showing MNX1-associated immune functions and pathways in CRC and LUAD, retrieved from BEST database. c, d) Knockdown efficiency of MNX1 at mRNA (c) and protein (d) levels in KYSE150 cells transfected with MNX1 siRNA or negative control siRNA, as confirmed by RT-qPCR and western blotting. The expression of PD-L1 was also detected by western blotting (d). The data are presented as mean ± SD (*n* = 3). The *p* value was calculated by one-way ANOVA. ***p* < 0.01.

**Figure S2**


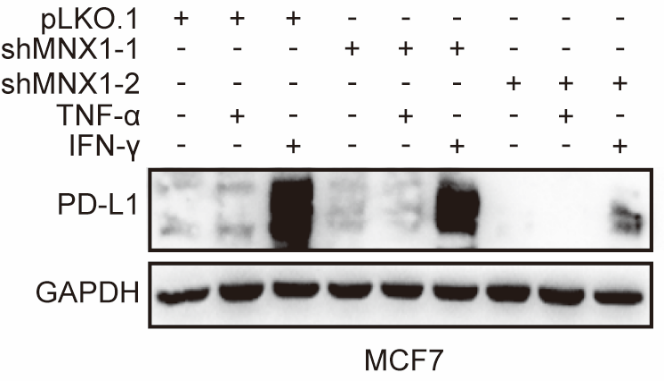


The protein levels of PD-L1 in MNX1-depleted MCF7 cells and control cells stimulated with TNF-α (20 ng/mL) or IFN-γ (20 ng/mL) for 12 h, as determined by western blotting.

**Figure S3**


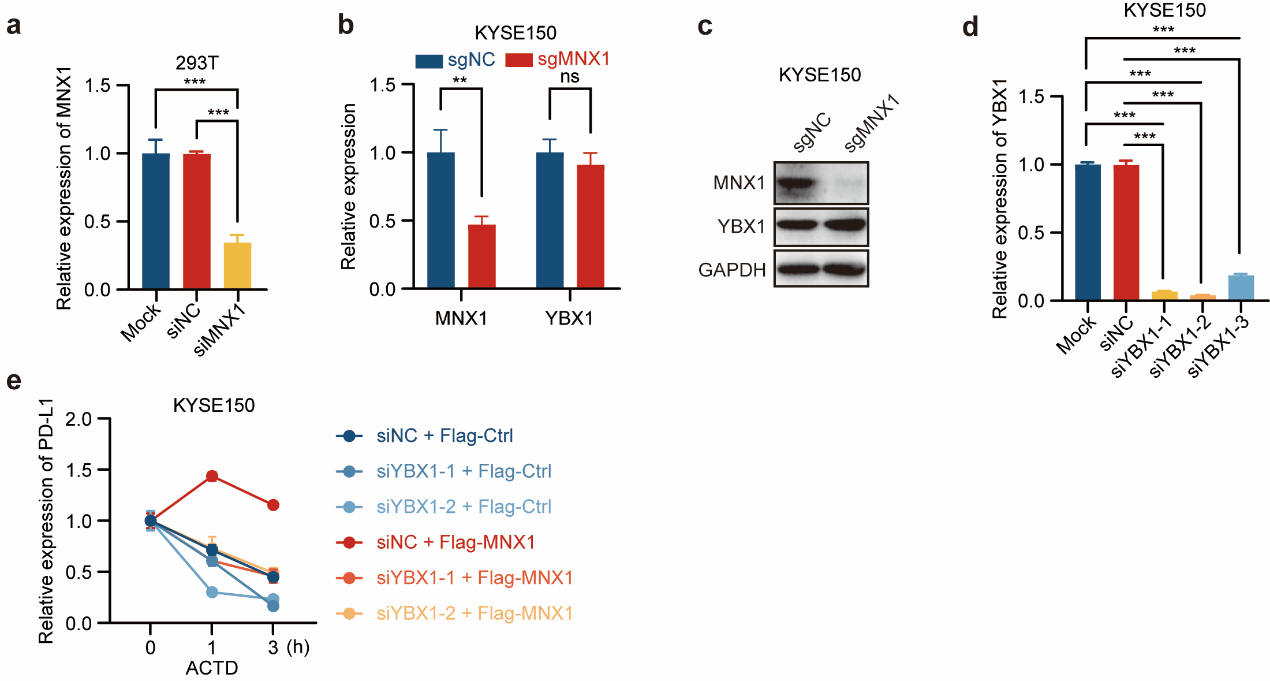


a) Knockdown efficiency of MNX1 in HEK293T cells transfected with MNX1 siRNA or negative control siRNA, as detected by RT-qPCR. b, c) The mRNA (a) and protein (b) levels of MNX1 and YBX1 in MNX1-KO KYSE150 cells or control cells, as determined by RT-qPCR and western blotting. d) Knockdown efficiency of YBX1 in KYSE150 cells transfected with YBX1 siRNAs or negative control siRNA, as detected by RT-qPCR. e) PD-L1 mRNA levels in KYSE150 cells transfected with YBX1 siRNA and Flag-tagged MNX1 vector successively and then treated with actinomycin D (ACTD; 5 μg/mL) for the indicated time points, as detected by RT-qPCR. The data are presented as mean ± SD (*n* = 3). The *p* value was calculated by two-tailed unpaired Student’s *t*-test in (b) and by one-way ANOVA in (a, d). ***p* < 0.01; ****p* < 0.001; ns, no significant.

**Figure S4**


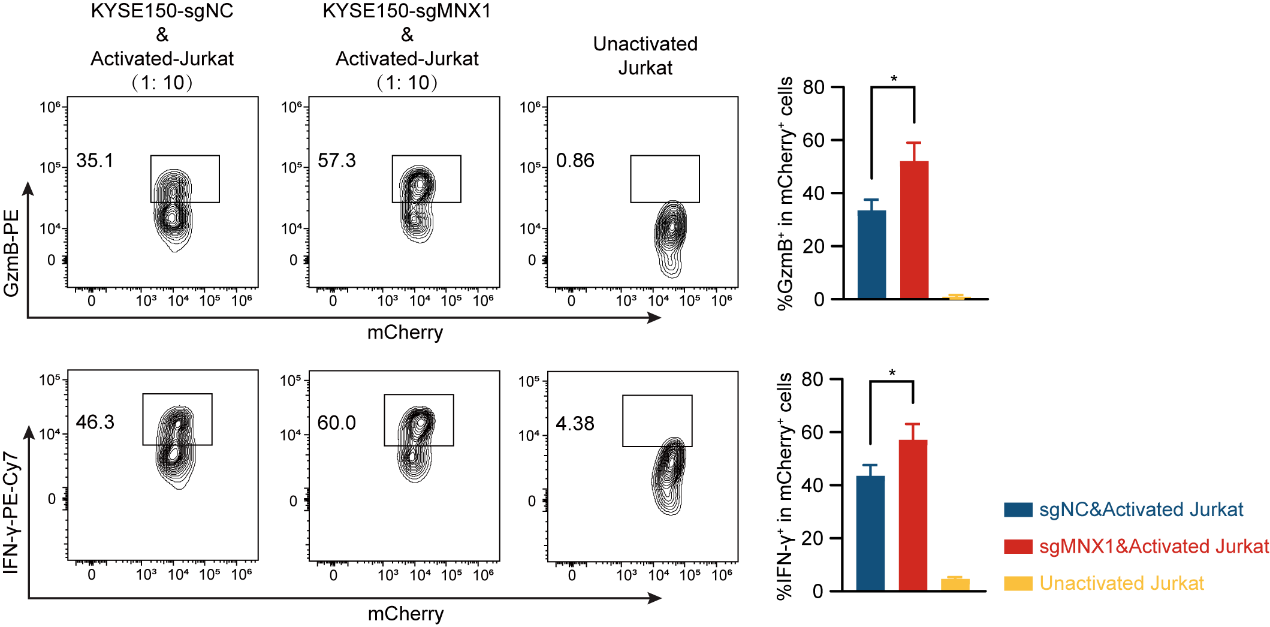


Flow cytometry analysis of GzmB and IFN-γ production in Jurkat cells activated and co-cultured with either MNX1-KO KYSE150 cells or control cells. The data are presented as mean ± SD (*n* = 3). The *p* value was calculated by two-tailed unpaired Student’s *t*-test. **p* < 0.05.

**Figure S5**


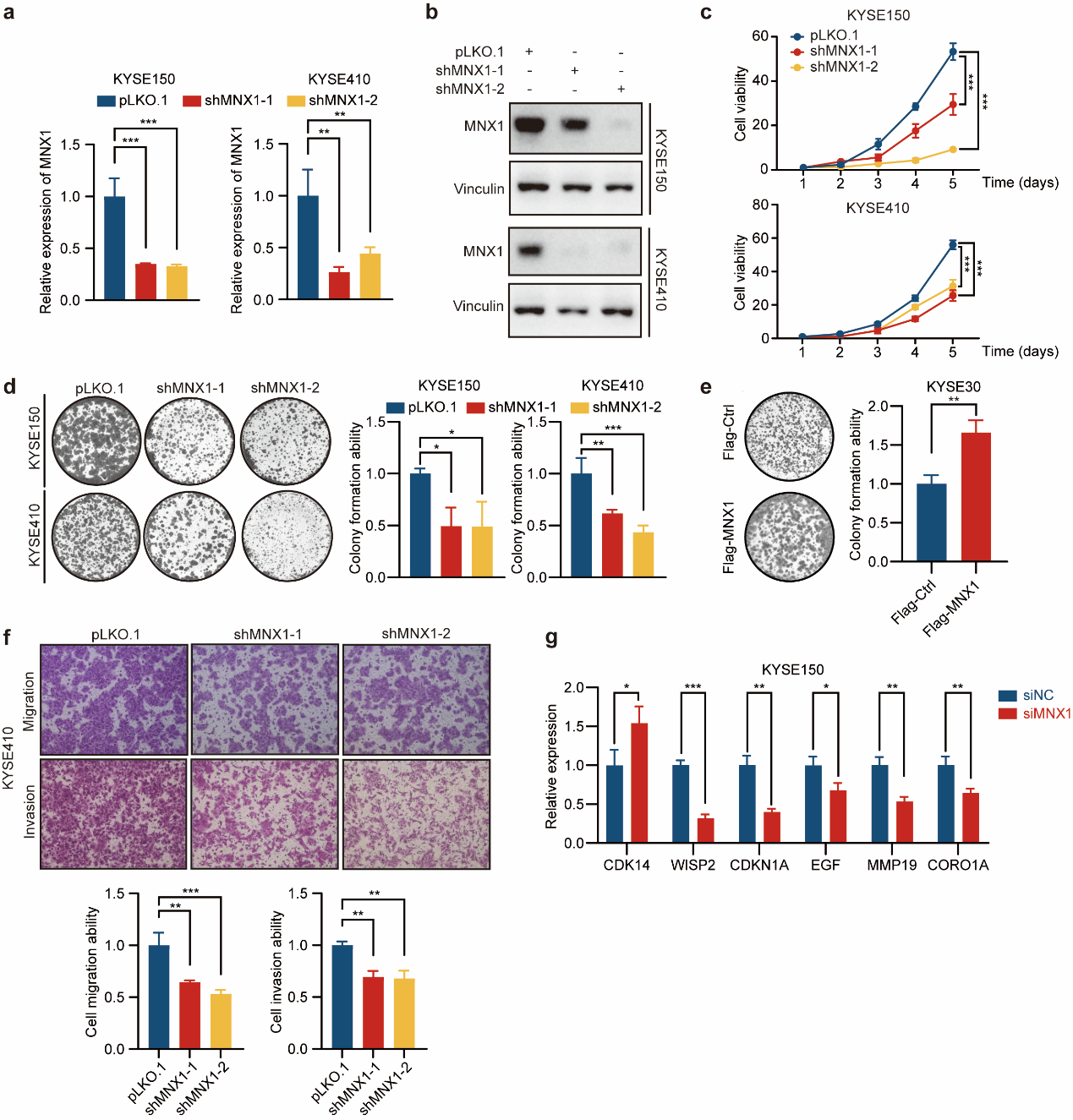


a, b) shRNA-mediated knockdown efficiency of MNX1 in KYSE150 and KYSE410 cells, as confirmed by RT-qPCR and western blotting. c) Growth curves of KYSE150 and KYSE410 cells upon MNX1 knockdown, as determined by CCK-8 assays. *n* = 5. d, e) Colony formation ability of ESCC cells upon MNX1 knockdown (d) or overexpression (e), as indicated. Representative images (left) and quantitative analyses (right) are shown. f) Migration and invasion abilities of KYSE410 cells upon MNX1 knockdown, as determined by Transwell migration and invasion assays. Representative images (top) and quantitative analyses (bottom) are shown. g) The mRNA levels of cell cycle and motility-related genes in KYSE150 cells transfected with MNX1 siRNA or negative control siRNA, as determined by RT-qPCR. The data are presented as mean ± SD (*n* = 3). The *p* value was calculated by two-tailed unpaired Student’s *t*-test in (e and g), by one-way ANOVA in (a, d, and f), and by two-way ANOVA in (c). **p* < 0.05; ***p* < 0.01; ****p* < 0.001.

**Figure S6**


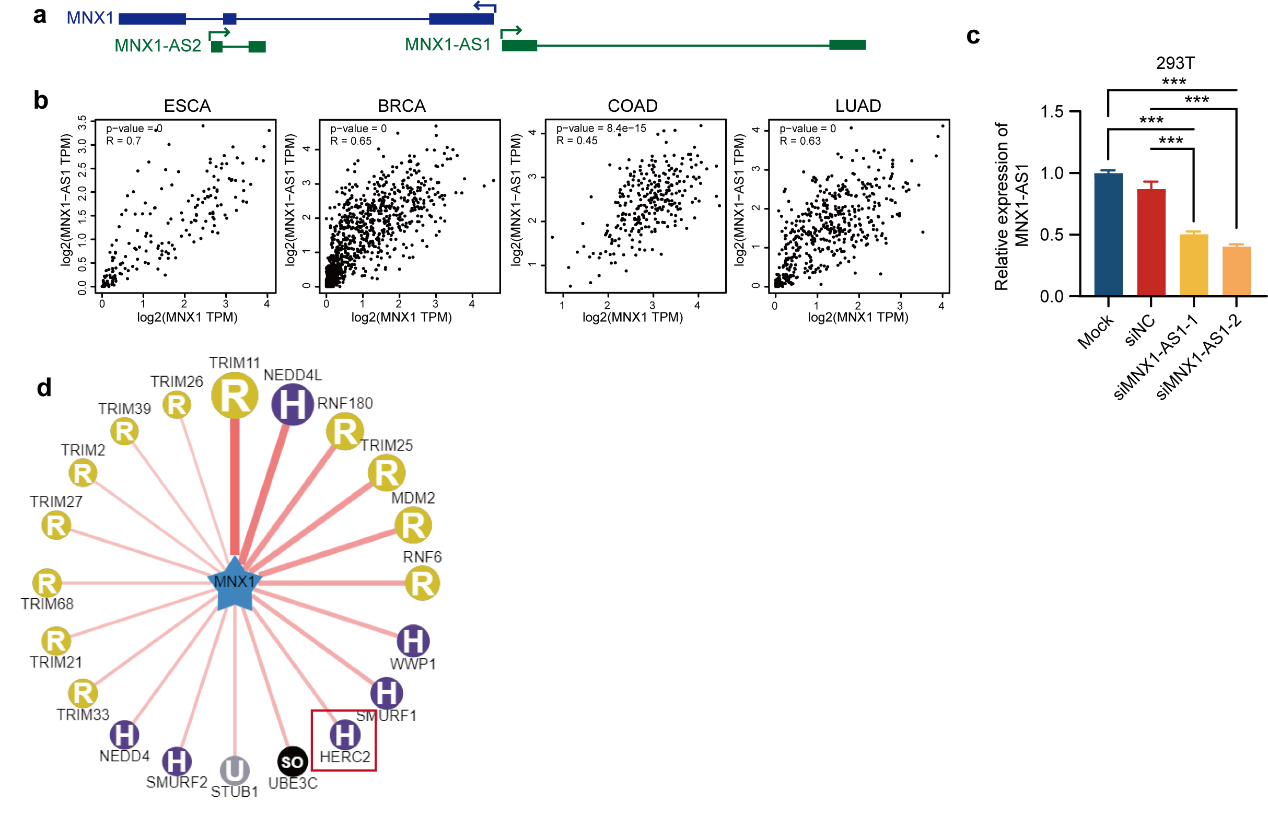


a) Position relationship between MNX1 and MNX1-AS1, based on UCSC Genome Browser (http://genome.ucsc.edu)[1]. b) Expression correlation of MNX1 mRNA and MNX1-AS1 in ESCA, BRCA, colon adenocarcinoma (COAD), and LUAD, obtained from GEPIA database. c) Knockdown efficiency of MNX1-AS1 in HEK293T cells transfected with MNX1-AS1 siRNAs or negative control siRNA, as detected by RT-qPCR. The data are presented as the mean ± SD (*n* = 3). The *p* value was calculated by one-way ANOVA. ****p* < 0.001. d) Predicted E3 ligases of MNX1, obtained from UbiBrowser database.

**References**

[1] B. J. Raney, G. P. Barber, A. Benet-Pages, J. Casper, H. Clawson, M. S. Cline, M. Diekhans, C. Fischer, J. Navarro Gonzalez, G. Hickey, A. S. Hinrichs, R. M. Kuhn, B. T. Lee, C. M. Lee, P. Le Mercier, K. H. Miga, L. R. Nassar, P. Nejad, B. Paten, G. Perez, D. Schmelter, M. L. Speir, B. D. Wick, A. S. Zweig, D. Haussler, W. J. Kent, M. Haeussler, *Nucleic Acids Res.* **2024**, *52*, D1082,
